# Group Psychosocial Interventions Following Acquired Brain Injury: A Systematic Review and Meta-analysis of Group Process and Outcomes

**DOI:** 10.1007/s11065-025-09670-w

**Published:** 2025-08-21

**Authors:** Hannah L. McGlashan, Kate Thompson, Michael Lam, Tegan Cruwys, Zoe C. Walter, Elizabeth Beadle, Catherine Haslam

**Affiliations:** 1 School of Psychology, The University of Queensland, Brisbane, QLD Australia; 2 Psychology Department, Royal Brisbane & Women’s Hospital, Brisbane, QLD Australia; 3 Centre for Advanced Imaging, The University of Queensland, Brisbane, QLD Australia; 4 School of Public Health, The University of Queensland, Brisbane, QLD Australia; 5 School of Medicine and Psychology, The Australian National University, Canberra, Australia; 6 Surgical Treatment and Rehabilitation Service, Metro North Hospital and Health Service, Brisbane, QLD Australia

**Keywords:** Psychosocial, Acquired brain injury, Rehabilitation, Group process

## Abstract

Acquired brain injury can result in disability with direct and indirect consequences for psychosocial functioning. Psychosocial interventions embedded within traditional neurorehabilitation may provide a valuable buffer. While there is evidence of benefits associated with group-based psychosocial interventions, there is no single recommended intervention, despite several different approaches having been trialled. This systematic review aimed to provide a critical appraisal of existing *group* psychosocial interventions in neurorehabilitation, meta-analyse their efficacy, and explore the contribution of group process to outcomes. Eligible studies were published in English-language peer-reviewed journals and recruited adults with acquired brain injury in receipt of group psychosocial interventions. Outcomes of interest were depression, anxiety, quality of life, emotional distress, community integration, and social support. A systematic search of CINAHL, PsycINFO, Medline, Web of Science and Embase from database inception until 08.07.2024 was conducted. Risk of bias was assessed using the Effective Public Health Practice Project Quality Assessment Tool for Quantitative Studies. Effect sizes were calculated using Hedges’ *g* and estimated using a three-level random effects model. Sixty-five studies were included in the systematic review, and 48 were included in the meta-analysis (*n* = 2653). There was an overall small effect on psychosocial outcomes (Hedges’ *g* = 0.24, 95% CIs [0.16, 0.33]), though none of the included studies satisfactorily analysed group process despite 70% of these studies proclaiming their importance. Overall, there were mixed findings across intervention type and significant heterogeneity. Recommendations for future psychosocial group interventions in neurorehabilitation are provided.

## Introduction

Acquired brain injury (ABI)—encompassing traumatic brain injury (TBI), stroke, brain tumours, infections, and hypoxia—can result in physical, cognitive, and psychological sequelae of varying severity depending on the location and extent of the injury. ABI represents a major cause of disability, imposing considerable economic and social costs by disrupting independent living, vocational outcomes, and community integration (Hart et al., 2011; Mahar & Fraser, 2020; Rapport et al., 2008). Rehabilitation approaches for ABI are typically informed by the biopsychosocial model, which emphasises the integration of physical, cognitive, psychological, and social care to support holistic recovery (Williams et al., 2014; Wright et al., 2016). However, resource constraints in acute and subacute rehabilitation settings tend to focus on remediation of physical and cognitive deficits that hinder functional independence and safe discharge (Mann et al., 2023). This is often at the expense of psychological and social components of care, leaving individuals at increased risk for psychosocial challenges such as depression, social isolation, and occupational deprivation post-discharge (Glintborg & Hansen, 2016; Højgaard Nejst & Glintborg, 2024; Turner et al., 2008). The COVID-19 pandemic has further compounded these challenges, including exacerbated unemployment rates, reduced access to social support, and heightened feelings of loneliness among ABI survivors (Wilkie et al., 2021).

ABI survivors commonly report feeling unprepared to manage these psychosocial difficulties and express the need for support to connect meaningfully with community services to reduce isolation (Abrahamson et al., 2017; Cott, 2004; Finch et al., 2020). Group-based psychosocial intervention represents one approach to addressing these needs by targeting psychological and social deficits, fostering community connectedness, and supporting adjustment. Despite the growing use of such interventions, we know little about the overall efficacy in ABI rehabilitation or the mechanisms through which improvements are supported. The current review aims to address this gap by synthesising and evaluating the efficacy of *group-based* psychosocial interventions that exist in ABI rehabilitation. Additionally, it explores the proposed therapeutic ingredients the group modality provides.

### The Psychosocial Consequences of Acquired Brain Injury

ABI disrupts psychosocial functioning across multiple domains, including perceptions of community reintegration, availability of social support, quality of life, and psychological functioning (Breting et al., 2017). These psychosocial domains are not only related to each other but also to the physical and cognitive sequelae that arise from ABI (Hammond et al., 2019; Mann et al., 2023). For example, mobility impairments may restrict access to facilities in the community and participation in social activities and events (Mills et al., 2022; Williams & Willmott, 2012). Cognitive fatigue can limit participation in community activities and impact occupational engagement (Åkerlund et al., 2021). Changes in social skills and emotional dysregulation can lead to inappropriate emotional responses impacting personal and vocational relationships (Maggio et al., 2022). Furthermore, the cognitive and physical impairments resulting from brain injury may be a source of social stigma and discrimination, causing individuals to withdraw from social situations (Bracho & Salas, 2024). Such social disruption, which increases the risk of loneliness, has been found to be a key predictor of subsequent mental health (Douglas, 2020; Morton & Wehman, 1995; Salas et al., 2022). It has been suggested that these psychosocial consequences might differ as a function of brain injury severity. For instance, individuals with mild injuries may experience temporary psychosocial difficulties before returning to baseline functioning within weeks to months (Broshek et al., 2015). However, 1 in 5 of these individuals and those with “complicated” mild injuries (i.e. those with positive neuroimaging findings) can experience similar cognitive and functional outcomes to those with injuries of moderate severity (Cancelliere & Mohammed, 2019; Kashluba et al., 2008). Given this, some authors have argued that early psychosocial intervention is recommended in individuals with any injury, irrespective of its severity (Batchos et al., 2018; Cancelliere et al., 2023). Added to this, individual differences such as age, pre-injury mental health, coping style, and resilience can also play a role in psychosocial outcomes (Scholten et al., 2020).

Considering the disruption to psychosocial function, it is not surprising that up to 60% of individuals with mild ‘complicated’ to severe TBI, and one-third of stroke survivors develop a psychiatric disorder within the first year post-injury (Ferro et al., 2016; Gould et al., 2011), with subclinical symptoms affecting even more individuals (Baldo et al., 2021). These challenges are associated with reduced quality of life, community integration, and employability, as well as increased mortality (Draper & Ponsford, 2008; Franulic et al., 2004; Ponsford et al., 2008). Concerningly, suicide risk is elevated among ABI populations, with TBI and stroke survivors having double and 73% higher risk, respectively, compared to the general population (Madsen et al., 2018; Vyas et al., (Vyas 2021)).

Despite the prevalence of psychosocial dysfunction in ABI populations and growing acknowledgement of the need to manage emotional health and well-being following brain injury, under-resourcing of rehabilitation services continues to undermine the provision of psychosocial intervention (Burke et al., 2020). Ultimately, the long-term impacts of psychosocial dysfunction result not only in costs to the individual and their family (Rigby et al., 2009) but also to government health and social services through reduced employment and increased social and primary care needs (Saka et al., 2009).

### Psychosocial Interventions in ABI Rehabilitation

Previous systematic reviews of psychosocial interventions have highlighted the potential benefits of tailored approaches but underscore methodological limitations and variability in treatment outcomes. Cattelani, et al., 2010 reviewed 63 intervention studies (*n* = 1094) and found that comprehensive-holistic rehabilitation programs were most effective for improving psychosocial functioning, while applied behaviour analysis and cognitive behaviour therapy (CBT) also showed benefit. Similarly, Davies and colleagues (2024) found combined cognitive and psychological interventions demonstrate small-to-medium effect sizes (*g* = 0.42) in improving quality of life and participation, with sustained benefits at follow-up. Another review by Verberne and colleagues (2019) found tailored CBT was effective for reducing anxiety, while behavioural management techniques reduced aggression/agitation. These authors also noted that recent studies have adopted stronger designs to address earlier methodological criticisms. Collectively, these reviews highlight the benefits of tailored and combined psychosocial interventions for ABI, while emphasising the need for robust methodologies to address variability and confounding factors.

The present review builds on this existing evidence-base by focusing specifically on *group-based* psychosocial interventions in ABI rehabilitation. Following a systematic search, we examined the literature to understand how “psychosocial interventions” have been previously categorised. However, we found that existing categorisations did not adequately capture the breadth and diversity of interventions identified in our search. To address this gap, we categorised interventions by systematically examining the core features and objectives of each intervention identified in our search and grouping them according to their predominant focus. The resulting framework tried to balance conceptual clarity and practical utility and comprised four approaches—notably, *neurorehabilitation psychology*, *activity-based intervention*, *community integration programs*, and *social skills training*.

Neurorehabilitation psychology is defined in the literature as an approach that “…supports, encourages, and maximises the processes of healthy development, adaptation, recovery, repair, quality of life, and adjustment” across the lifespan for persons with neurological conditions (Stucky et al., 2024, p. 605). Interventions are dynamic and varied, incorporating both psychotherapeutic and cognitive neurorehabilitation techniques. Psychotherapeutic techniques may include cognitive behaviour therapy (CBT; Beck, 1979), mindfulness-based stress reduction (MBSR; Kabat-Zinn, 1990), mindfulness-based cognitive therapy (MBCT; e.g. Segal, 2002) and other evidence-based psychological approaches. Cognitive neurorehabilitation techniques include cognitive strategies, psychoeducation, and structured approaches such as goal management training or problem-solving therapy.

Activity-based interventions foster social interaction and the formation of meaningful supportive relationships among individuals sharing mutual interest, thereby enhancing wellbeing in ABI populations (Tate et al., 2014). Activities can include: group-based exercise and leisure programs, which are designed to increase physical activity and participation through peer interaction and support (Mitchell et al., 2014); art-based therapy, encompassing activities like painting, sculpture, dance, and storytelling, which has garnered increasing recognition for its role in promoting psychological and social recovery within healthcare settings (Barker & Brunk, 1991); and music-making activities, used to integrate physical, psychological, and social elements of rehabilitation, with established psychosocial benefits including emotional regulation and expression, social connection, and identity (Rosado & Vaudreuil, 2023).

Social skills training programs include strategies for social communication, self-monitoring of speech and language, social behaviour (i.e. awareness of social rules and boundaries) and emotional control, with the aim of improving social competence (Dahlberg et al., 2007). Social skills training programs often utilise the group modality to incorporate group feedback and social interaction (Yalom & Leszcz, 2005). However, the evidence base exploring social skill interventions is limited, making it difficult to determine a single best form of delivery (Braden et al., 2010).

Community integration programs focus on enabling individuals to participate meaningfully in social, occupational, recreational, and educational activities while addressing any underlying physical, cognitive, emotional, or social challenges that arise after injury (McCabe et al., 2007). They often include aspects of self-management where the aim is to teach patients to take an active role in managing their health conditions and support problem-solving, goal setting, and accessing resources in the community with the overarching aim to build self-efficacy (Kim & Colantonio, 2010).

### Group-Based Psychosocial Interventions

The efficacy of psychosocial interventions is influenced not only by their content but also by their delivery method. While traditionally delivered one-on-one, group-based modalities are increasingly utilised due to their practical and therapeutic advantages. Group programmes are cost and time-efficient, allowing greater therapist availability and reducing wait times for accessing services (Aboulafia-Brakha et al., 2013). This is especially true for specialist trained staff (e.g. psychologist/neuropsychologist) who are increasingly acknowledged as a core discipline in rehabilitation, but often inadequately staffed within services (Glen et al., 2020; Sweet et al., 2021; Ta’eed et al., 2015). Moreover, group psychotherapy stands as a recognised method in holistic care, demonstrating therapeutic efficacy comparable to many forms of individual therapy (McRoberts et al., 1998; Tucker & Oei, 2007). It is less clear, however, whether group therapy is comparable to individual therapy across other psychosocial intervention types.

Beyond practical advantages, the group modality holds potential *additive* benefits for therapeutic outcomes. Preliminary work suggests that social groups—including therapeutic groups—provide access to a variety of psychological resources (Cruwys et al., 2020; Haslam et al., 2016, 2018; Steffens et al., 2021; Tarrant et al., 2020). For instance, within a supportive environment, group interventions can improve self-efficacy, emotional well-being, access to social support, and reduce feelings of illness-related stigma (Haslam et al., 2008, 2010; Vickers, 2010). Additionally, peer feedback, interaction, and support fostered in group settings may facilitate the development of social skills and alleviate feelings of social isolation, thus promoting community integration (Brabender et al., 2004). Beyond individual therapy, group interventions can normalise experiences and enhance self-awareness (Patterson et al., 2016). Furthermore, they can cultivate a sense of community, even among more passive participants or those with limited communication skills (Gurr, 2009). Importantly, group rehabilitation is highly acceptable to individuals with brain injuries (Patterson et al., 2019).

The effectiveness of group interventions depends on fostering a sense of shared connectedness and group identification, which enable participants to work collectively toward treatment goals (Cruwys et al., 2014; Haslam et al., 2018; Robertson et al., 2023). According to the social identity approach to health, shared group identification predicts better treatment engagement and outcomes. This is corroborated by a recent meta-analysis of social identification-building group interventions, which demonstrated that the presence of group identification enhances treatment outcomes (Steffens et al., 2021). Research further highlights the impact of group identification on psychosocial outcomes, showing its role in reducing depression (Sani et al., 2012; Steffens et al., 2021) and post-traumatic stress (Muldoon & Downes, 2007), as well as facilitating adjustment to life change—such as recovery from addiction (Dingle et al., 2015) and stroke (Haslam et al., 2008). Within group interventions themselves, the role of group processes, such as cohesion (i.e. feelings of connectedness and emotional safety within therapy groups), has been proposed as key mechanisms through which therapeutic benefits are achieved (Burlingame & Jensen, 2017). Taken together, these findings underscore the necessity of intentionally incorporating group processes into the design and implementation of interventions. For group-based approaches to optimally improve psychosocial outcomes, consideration of factors such as group identification and cohesion must be prioritised, rather than left to chance.

Group-based approaches also have limitations. Practical challenges include rigid scheduling and reduced opportunities for individualised treatment (Hammond et al., 2015). Additionally, group mechanisms, such as cohesion, require skilled facilitation (Burlingame & Jensen, 2017). Moreover, group heterogeneity in factors such as injury severity, aetiology, and functioning can disrupt dynamics, necessitating careful consideration of group composition to optimise outcomes (Hammond et al., 2015; Patterson et al., 2016). These challenges necessitate additional attention to ensure group delivery of interventions is relevant and meaningful and might require the creation of separate patient groups based on injury severity and aetiology.

### Present Research

Psychosocial concerns after brain injury are abundant but are not currently prioritised in rehabilitation (Jackson et al., 2020; Mann et al., 2023). There is emerging evidence suggesting group-based psychosocial interventions have the potential to improve psychosocial outcomes, but the approaches are varied and little is known about overall efficacy (Cicerone et al., 2019; Hammond et al., 2015). The present review builds on previous literature that has established the potentiality of psychosocial interventions in ABI rehabilitation by focusing on group-based programs. More specifically, this review aimed to (a) identify the range of group psychosocial interventions used in ABI rehabilitation, (b) evaluate their impact on psychosocial outcomes, and (c) examine how group processes contribute to these outcomes.

## Method

This review followed the Preferred Reporting Items for Systematic Reviews and Meta‐Analyses (PRISMA) best practice guidelines (Page et al., 2021; see Online Resource 1) and the protocol was prospectively registered with the International Prospective Register of Systematic Reviews (PROSPERO, registration no. CRD42021238920). Given the lack of a single accepted definition of “psychosocial function” or “psychosocial intervention” (Gonzalez et al., 2015), the authorship team developed a consensus definition for this review. Our definition of psychosocial function was informed by the neuropsychological rehabilitation literature (Gracey et al., 2009; Honan et al., 2019; Morton & Wehman, 1995; Wilson et al., 2009) that recognises the contributions of both internal psychological functioning (reflected in one’s mental health, inclusive of anxiety, depression, and emotional distress) and external community functioning (reflected in one’s sense of community integration and available support) with quality of life being captured in both aspects.

During the systematic review, it became evident that while some heterogeneity existed across studies meeting the inclusion criteria, the data were sufficiently similar to warrant a meta-analysis. To account for the identified heterogeneity and ensure a robust synthesis, the original protocol was extended to include a three-level meta-analysis, offering a robust method to pool data and explore variability systematically. The data and analysis code for the meta-analysis are included as supplementary materials and are available via the Open Science Framework: https://osf.io/efbqk/?view_only=28e928d2a1e64e7f86dd03bfe423fc6d.

### Defining Psychosocial Outcomes

The review outcome measures deviated slightly from the original protocol because the search revealed over 100 different outcome measures spanning 23 distinct “psychosocial” constructs. This breadth highlights the heterogeneity in defining psychosocial functioning within this field. To refine the scope and ensure a focused analysis, our selection of relevant outcome measures was guided by the framework developed by the Australian National Health and Medical Research Council’s (NHMRC) “Moving Ahead” Centre of Research Excellence (CRE) in Brain Recovery (Honan et al., 2019). Given the framework’s extensive nature, a comprehensive examination of all identified aspects was beyond the scope of this review. Therefore, to provide a clearer focus, the research team selected six of the most consistently measured and theoretically grounded psychosocial constructs in the brain injury intervention literature for analysis:

1. Anxiety: Anxiety is one of the most prevalent psychological sequelae of ABI, characterised by excessive worry, heightened physiological arousal, and increased fearfulness, which can impede recovery and rehabilitation outcomes (Rafsten et al., 2018).
2. Depression: Depression is another common post-ABI psychological condition, marked by persistent feelings of sadness, loss of interest or pleasure, and reduced motivation, which can hinder rehabilitation progress and impact overall quality of life (Scholten et al., 2016).
3. Emotional distress: This construct represents a broader range of neurobehavioural and affective difficulties experienced post-ABI, rather than discrete psychological disorders (Shields et al., 2016). This review included outcome measures defined within the respective studies as overarching measures of psychological or emotional distress, psychological wellbeing, or emotional dysregulation. This also encompassed composite scores from anxiety and depression scales when reported as a single, overall measure of distress.
4. Quality of life: This is a very common outcome measure in ABI research, capturing the broad impact of the injury on an individual’s physical, emotional, and social well-being (Dua et al., 2024)
5. Community integration: This construct is a key indicator of successful rehabilitation, reflecting an individual’s capacity to reintegrate into work, social, and recreational activities (Proctor & Best, 2019; Williams et al., 2014).
6. Social support: A multidimensional construct encompassing emotional, practical (e.g. tangible assistance), and informational support. It can be assessed subjectively (perceived support), objectively (received support), or by examining the extent of an individual’s social connectedness. Strong support is essential for mitigating the adverse effects of ABI, positively influencing mood, wellbeing, and coping mechanisms, thereby contributing to long-term recovery (Batchos et al., 2018; Harandi et al., 2017; Salas et al., 2022).

**Fig. 1:**
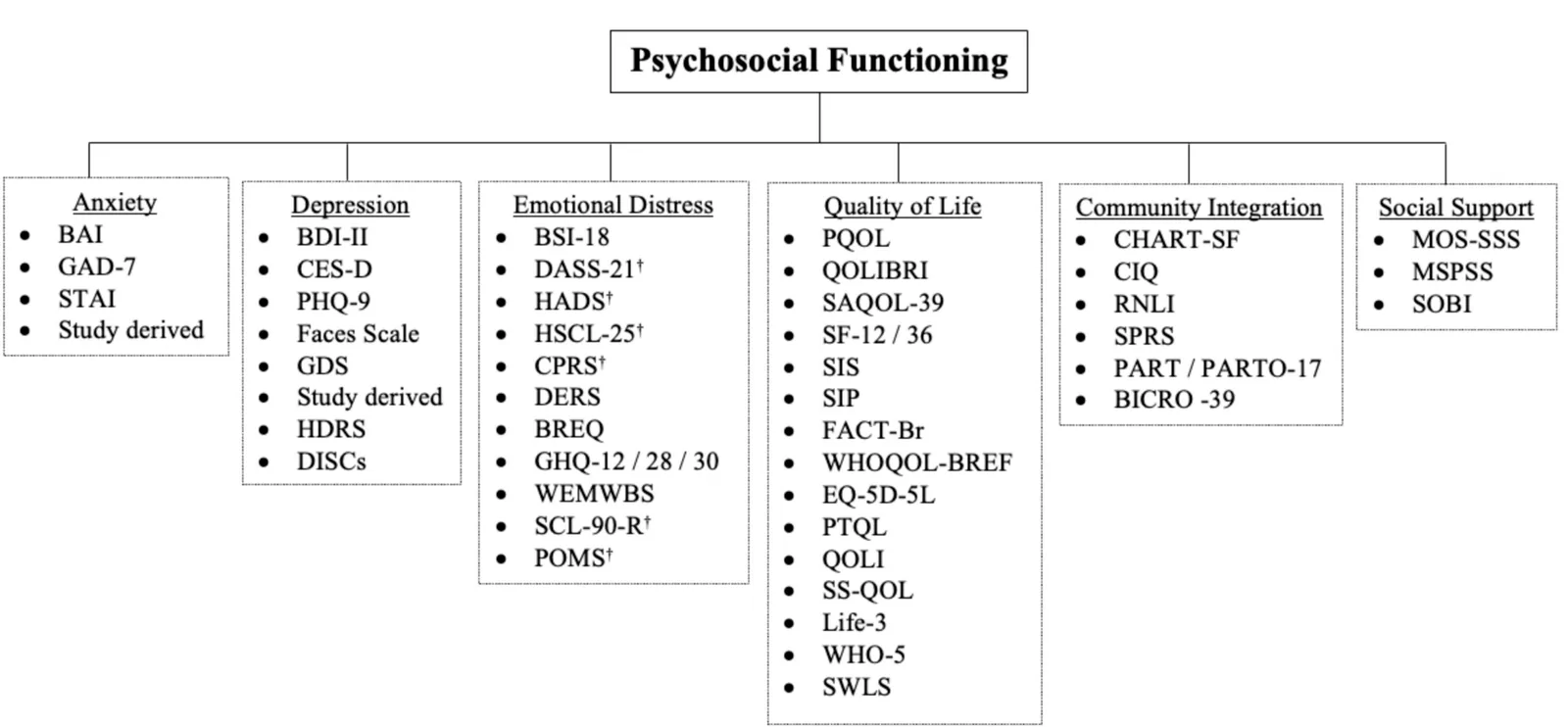
Psychological constructs and associated outcome measures used in the current review. ^†^Subscales used to measure anxiety and/or depression. *HADS* = Hospital Anxiety and Depression Scale (Zigmond & Snaith, 1983); *SF-36* = 36-item Short-Form Health Survey (Ware & Sherbourne, 1992); *SIP* = Sickness Impact Profile (Bergner et al., 1976); *WHOQOL* = World Health Organization Quality of Life (WHOQOL Group, 1998); *DASS-21* = Depression Anxiety and Stress Scales (Lovibond & Lovibond, 1995); *CIQ* = Community Integration Questionnaire (Willer et al., 1993); *PQOL* = Perceived Quality of Life (Cicerone & Azulay, 2007); *BSI-18* = Brief Symptom Inventory (Derogatis, 2001); *GDS* = Geriatric Depression Scale (Sheikh & Yesavage, 1986); *STAI* = State-Trait Anxiety Inventory (Spielberger et al., 1983); *CES-D* = Centre for Epidemiologic Studies Depression Scale (Radloff, 1977); *BDI-II *= Beck Depression (Beck et al., 1996); *PHQ* = Patient Health Questionnaire (Kroenke et al., 2001); *SCL-90-R* = Symptom Checklist-90-Revised (Derogatis, 1994); *DISCs* = Depression Intensity Scale Circles (Turner-Stokes et al., 2005); *PART* = Participation Assessment with Recombined Tools (Bogner, 2013); *SS-QOL* = Stroke-Specific Quality of Life Scale (L. S. Williams et al., 1999); *QOLIBRI* = Quality of Life after Brain Injury (von Steinbüchel et al., 2010); *CHART-SF* = Craig Handicap Assessment and Reporting Technique- Short Form (Whiteneck et al., 1992); *SPRS* = Sydney Psychosocial Reintegration Scale (Tate et al., 1999); *BICRO-30* = Brain Injury Community Rehabilitation Outcome scales (Powell et al., 1998); *MOS-SSS* = Medical Outcomes Study – Social Support Survey (Sherbourne & Stewart, 1991); *RNLI* = Reintegration to Normal Living Index (Wood-Dauphinee et al., 1988); *CPRS* = Comprehensive Psychopathological Rating Scale (Svanborg & Asberg, 1994); *PTQ*L = Pictorial Thai Quality of Life (Phattharayuttawat et al., 2005); Life-3 (Andrews & Withey, 1976); *SIS* = Stroke Impact Scale (Duncan et al., 1999); *HDRS* = Hamilton Depression Rating Scale (Hamilton, 1960); *SF*-12 = Short Form Health Survey – 12 items (Ware et al., 1996); *PHQ-9* = Patient Health Questionnaire (Kroenke et al., 2001); *GAD-7* = Generalised Anxiety Disorder Assessment—7 item (Spitzer et al., 2006); EQ-5D-5L (Herdman et al., 2011); *BAI* = Beck Anxiety Inventory *MSPSS* = Multidimensional Scale of Perceived Social Support (Zimet et al., 1988); *SOBI* = Sense of Belonging Instrument (Hagerty & Patusky, 1995); *SAQOL-39* = Stroke and Aphasia Quality of Life Scale (Hilari et al., 2003); *QOLI* = Quality of life inventory (Frisch, 1994);* HSCL-25* = The Hopkins Symptom Checklist-25 (Derogatis et al., 1974); *FACT-Br* = Functional Assessment of Cancer Therapy – Brain (Thavarajah et al., 2013); Faces Scale (McDowell, 2006); *DERS* = Difficulties in Emotion Regulation Scale (Gratz & Roemer, 2004); *BREQ* = Brain Injury Rehabilitation Trust Regulation of Emotions Questionnaire (Cattran et al., 2011); *GHQ* = General Health Questionnaire (Goldberg & Hillier, 1979); *WEMWBS* = Warwick-Edinburgh Mental Well-being Scale (Tennant et al., 2007); *POMS* = Profile of Mood States (McNair et al., 1971); *WHO-5* = The World Health Organisation-Five Well-Being Index (World Health Organization, 2024); *SWLS* = Satisfaction with Life Scale (Diener et al., 1985)

Together, these constructs provide a comprehensive and meaningful framework for evaluating the psychosocial consequences of ABI, aligned with established recommendations for outcome measurement while maintaining a targeted and manageable scope. The selected constructs and the outcome measures included in this review are illustrated in Fig. 1.

### Eligibility Criteria

To be included in the systematic review, studies had to meet the following eligibility criteria:

- Types of studies: We included all published within or between-group experimental, cohort or quasi-experimental designs (we did not limit to randomised controlled trials), including pilot studies examining feasibility. Unpublished manuscripts, conference abstracts, and other grey literature were not eligible for inclusion. Language was restricted to English. We did not exclude studies based on the date of publication.
- Types of participants: We included adults (i.e. over the age of 18 years) of any sex with a diagnosis of acquired brain injury of any mechanism and severity (i.e. mild, moderate, and severe). We excluded studies in which participants presented with progressive neurological conditions (e.g. multiple sclerosis, and dementia). However, we did not exclude studies that recruited patients with health or psychiatric co-morbidities as these often co-occur in ABI populations.
- Types of interventions: Participants may have received the intervention in any setting including inpatient, outpatient, or community and may have received the intervention in addition to treatment as usual. The intervention had to be conducted in a group, and studies were excluded if the intervention included group plus individual sessions. The intervention had to be described as psychosocial, which was defined in this paper as a program designed to produce change in a participant’s psychological functioning, and/or social functioning, and/or global well-being. We included studies with or without a control group.
- Types of outcome measures: This comprised any scale that was reported in the study to measure depression, anxiety, emotional distress, quality of life, community integration, or social support (see Fig. 1). We included study-developed scales in addition to validated measures of these constructs. Where data were reported separately, subscales within broader measures were also included. We excluded all other psychological, cognitive, and functional outcomes.

Additional criteria for inclusion in the meta-analysis required that studies employed either a single group pre-post intervention design, or an independent groups pre-post intervention design. Furthermore, studies had to provide sufficient pre- and post-intervention data within the manuscript or from the authors to perform effect size analyses for the outcomes of interest (e.g. means and standard deviations, inferential statistics such as *t*, or *F* values, or change scores). To assess statistical heterogeneity, we used Cochran’s *Q* test and the *I*^2^ statistic. The results indicated moderate heterogeneity, with an *I*^2^ value below 50% considered indicative of acceptable homogeneity for pooling data. Additionally, we conducted moderator analyses based on (1) the type of intervention and (2) the outcome measured. These analyses demonstrated that variations in outcome contributed to some of the observed heterogeneity. Taken together, these criteria and analyses ensured that the included studies were sufficiently homogenous to conduct a meta-analysis.

### Search Strategy

The comprehensive search strategy was developed in consultation with a health sciences librarian and adapted for five electronic databases: CINAHL, PsycINFO, Medline, Web of Science and Embase. Searches were from inception with search alerts set. The original search was conducted on 11/03/2021 and a final search update was completed on 08/07/2024. The following combinations of three components were used in the search strategy: (“brain injury” OR “acquired brain injury” OR “traumatic brain injury” OR “ABI” OR “TBI” OR “stroke”) AND (“neuropsychological rehabilitation” OR “psychosocial” OR “cognitive rehabilitation” OR “neuro-rehabilitation” OR “neurorehabilitation” OR “psychosocial rehabilitation” OR “psycho-social rehabilitation” OR “holistic rehabilitation” OR “multidisciplinary rehabilitation” OR “cognitive behaviour therapy” OR “cognitive behavior therapy”) AND ((title: “group*”) OR “group intervention” OR “group cohesion” OR “group dynamics” OR “peer groups” OR “group treatment” OR “programme”). The term “group” was limited to title searches only due to yielding too many unrelated results on scoping searches. In addition, we searched the reference lists of all eligible studies (backward chaining) and undertook forward citation chaining (using Google Scholar) to identify further eligible studies. See Online Resource 2 for more details of the search strategy.

### Data Extraction

For all included studies, data was extracted by two authors (HM and KT). Inter-rater agreement was almost perfect for both the meta-analysis data at 99.2% (*κ* = 0.98, 95% CI, 0.97–0.98) and the methodological data at 98.3% (*κ* = 0.97, 95% CI, 0.95–0.98). These data are summarised in Table 1.

**Table 1:** Extracted data from all studies

Study	Intervention type	*N*	Design	Effect size (Hedges’ g)	Population	Country	Duration (session length)/intensity	Psychosocial outcome (measure)
Aboulafia-Brakha et al. (2013)	Neurorehabilitation psychology	10	SGPPD (Cohort)	n/a	TBI	Switzerland	8 weeks (60 min)/weekly	Anxiety (HADS-A) Depression (HADS-D) Quality of life (SF-36)
Anson et al. (2006a)	Neurorehabilitation psychology	33	SGPPD (Cohort)	n/a	TBI	Australia	5 weeks (90 min)/2 × per week	Anxiety (HADS-A) Depression (HADS-D) Quality of life (SIP)
Anson et al. (2006b)	Neurorehabilitation psychology	31	IGPPD (Cohort)	n/a	TBI	Australia	5 weeks (90 min)/2 × per week	Anxiety (HADS-A) Depression (HADS-D) Quality of life (SIP)
Appleton et al. (2011) Effect size a Effect size b Effect size c Effect size d Effect size e Effect size f	Social skills training	7	SGPPD (Cohort)	0.00 − 0.32 0.04 0.00 − 0.03 0.45	TBI (4), CVA (3)	Australia	4 weeks (90 min)/3 × per week	Anxiety (HADS-A) Depression (HADS-D) Quality of life (WHOQOL–Physical) Quality of life (WHOQOL–Psychological) Quality of life (WHOQOL–Social) Quality of life (WHOQOL–Environment)
Arundine et al. (2012) Effect size a Effect size b Effect size c	Neurorehabilitation psychology	17	IGPPD (Cohort analytic)	− 0.61 − 0.22 − 0.43	Trauma (9), non-trauma (8)	Canada	11 weeks (60–90 min)/weekly	Emotional distress (DASS-21) Emotional distress (SCL-90-R) Community integration (CIQ)
Azulay et al. (2013) Effect size a	Neurorehabilitation psychology	22	SGPPD (Cohort)	0.42	Mild TBI	USA	10 weeks (120 min)/weekly	Quality of life (PQOL)
Backhaus et al. (2010)	Neurorehabilitation psychology	20	IGPPD (RCT)	n/a	TBI (9), other (11)	USA	12 weeks (120 min)/weekly	Emotional distress (BSI-18)
Backhaus et al. (2016) Effect size a	Neurorehabilitation psychology	19	IGPPD (RCT)	− 0.52	TBI (6), other (13)	USA	16 weeks (120 min)/weekly	Emotional distress (BSI-18)
Baldo et al. (2021) Effect size a Effect size b Effect size c Effect size d Effect size e Effect size f Effect size g Effect size h Effect size i Effect size j Effect size k	Neurorehabilitation psychology	27	IGPPD (RCT)	− 0.34 − 0.28 − 0.32 0.15 − 0.32 − 0.18 − 0.11 − 0.54 − 0.32 − 0.28 0.43	CVA	USA	8 weeks (150 min)/weekly	Depression (GDS) Anxiety (STAI) Quality of life (SF-36–Physical) Quality of life (SF-36–Physical) Quality of life (SF-36–Psychological) Quality of life (SF-36–Fatigue) Quality of life (SF-36–Psychological) Quality of life (SF-36–Social) Quality of life (SF-36–Pain) Quality of life (SF-36–Health) Quality of life (SWLS)
Bay and Chan (2019) Effect size a	Neurorehabilitation psychology	25	IGPPD (Controlled clinical trial)	0.53	TBI	USA	8 weeks (90 min)/weekly	Depression (CES-D)
Bedard et al. (2003) Effect size a Effect size b Effect size c Effect size d Effect size e Effect size f Effect size g	Neurorehabilitation psychology	13	IGPPD (Cohort analytic)	0.91 1.39 0.56 − 0.31 0.05 − 0.16 0.45	TBI	Canada	12 weeks (not reported)/weekly	Depression (BDI-II) Quality of life (SF-36–Psychological) Quality of life (SF-36–Physical) Community integration (CIQ–Home) Community integration (CIQ–Social) Community integration (CIQ–Productivity) Emotional distress (SCL-90-R)
Bedard et al. (2014) Effect size a Effect size b Effect size c	Neurorehabilitation psychology	76	IGPPD (RCT)	0.60 0.07 0.06	TBI	Canada	10 weeks (90 min)/weekly	Depression (BDI-II) Depression (PHQ) Depression (SCL-90-R–Depression subscale)
Blake et al. (2024)	Neurorehabilitation psychology	117	SGPPD (Cohort)	n/a	CVA	UK	Open group (range of attended sessions 1 to 11)	Depression (DISCs)
Bradbury et al. (2008) Effect size a Effect size b Effect size c	Neurorehabilitation psychology	20	IGPPD (Cohort analytic)	0.22 0.82 0.82 n/a	Trauma (10), non-trauma (10)	Canada	9 weeks (45–75 min)/1–2 × per week (10 sessions total)	Depression (DASS-21) Anxiety (DASS-21) Emotional distress (SCL-90-R) Community integration (CIQ)
Braden et al. (2010)	Social skills training	30	SGPPD (Cohort)	n/a	TBI	USA	13 weeks (90 min)/weekly	Community integration (PART) Quality of life (SWLS)
Cassel et al. (2024) Effect size a Effect size b Effect size c	Social skills training	21	IGPPD (RCT)	0.54 0.68 0.66	TBI (18), ABI (3)	Australia	14 weeks (90 min)/weekly	Depression (HADS-D) Anxiety (HADS-A) Quality of life (QOLIBRI)
Chalmers et al. (2019) Effect size a Effect size b Effect size c	Neurorehabilitation psychology	29	IGPPD (Cohort analytic)	− 0.19 − 0.15 0.16	CVA	New Zealand	6 weeks (90 min)/weekly	Depression (CES-D) Anxiety (HADS-A) Quality of life (SS-QOL)
Combs et al. (2018) Effect size a Effect size b	Neurorehabilitation psychology	19	SGPPD (Cohort)	− 0.07 0.56	Trauma (15), non-trauma (4)	USA	32 weeks rolling attendance (60 min)/weekly	Anxiety (study derived measure) Depression (study derived measure)
Cuberos-Urbano (2018) Effect size a Effect size b Effect size c Effect size d Effect size e Effect size f	Neurorehabilitation psychology	16	IGPPD (Controlled clinical trial)	0.43 0.06 0.22 0.27 0.13 0.53	TBI (11), CVA (5)	Spain	7 weeks (60 min)/2 × per week	Quality of life (QOLIBRI–Cognition) Quality of life (QOLIBRI–Self) Quality of life (QOLIBRI–Autonomy) Quality of life (QOLIBRI–Social) Quality of life (QOLIBRI–Psychological) Quality of life (QOLIBRI–Physical)
Dahlberg et al. (2007) Effect size a Effect size b Effect size c Effect size d Effect size e	Social skills training	52	IGPPD (RCT)	− 0.09 0.33 − 0.04 0.06 0.08	TBI	USA	12 weeks (90 min)/weekly	Community integration (CIQ–Social) Community integration (CIQ–Productivity) Community integration (CHART-SF–Occupation) Community integration (CHART-SF–Social) Quality of life (SWLS)
Di Vita et al. (2020)	Activity-based	9	SGPPD (Controlled clinical trial)	n/a	TBI	Italy	Not reported	Quality of life (QOLIBRI) Depression (BDI-II) Anxiety (STAI-X) Emotional distress (DERS)
Donnelly et al. (2017) Effect size a	Activity-based	31	IGPPD (Cohort analytic)	0.69	Trauma (26), non-trauma (5)	USA	8 weeks (60 min)/2 × per week	Quality of life (QOLIBRI)
Donnelly et al. (2021) Effect size a	Activity-based	705	SGPPD (Cohort)	0.55	TBI	USA	6 weeks (90 min)/weekly	Quality of life (QOLIBRI)
Ellis-Hill et al. (2019) Effect size a Effect size b Effect size c Effect size d Effect size e Effect size f Effect size g Effect size h Effect size i Effect size j Effect size k	Activity-based	56	IGPPD (RCT)	0.42 0.23 0.17 − 0.01 − 0.14 0.13 0.02 0.28 0.10 0.20 0.10	CVA	UK	10 weeks (120 min)/weekly	Depression (HADS-D) Anxiety (HADS-A) Quality of life (SF-36–Physical) Quality of life (SF-36–Physical) Quality of life (SF-36–Psychological) Quality of life (SF-36–Fatigue) Quality of life (SF-36–Psychological) Quality of life (SF-36–Social) Quality of life (SF-36–Pain) Quality of life (SF-36–Health) Emotional distress (WEMWBS)
Faulkner et al. (2015) Effect size a Effect size b Effect size c Effect size d Effect size e	Activity-based	55	IGPPD (RCT)	0.65 0.01 0.19 0.06 0.16	TIA (33), mild stroke (22)	New Zealand	8 weeks (90 min)/2 × per week (exercise) + (30 min)/weekly (education)	Quality of life (SF-36–Physical) Quality of life (SF-36–Psychological) Depression (HADS-D) Anxiety (HADS-A) Depression (POMS–Depression subscale)
Fleming et al. (2009) Effect size a Effect size b Effect size c Effect size d Effect size e	Community integration	36	IGPPD (Cohort analytic)	0.76 0.38 0.07 − 0.04 0.24	TBI (21), CVA (5), other (10)	Australia	6 weeks (120 min)/weekly	Community integration (SPRS) Depression (HADS-D) Anxiety (HADS-A) Community integration (BICRO-30) Social support (MOS-SSS)
Gurr et al. (2009)	Neurorehabilitation psychology	34	SGPPD (Cohort)	n/a	CVA	UK	2 weeks (90 min)/weekly	Anxiety (HADS-A) Depression (HADS-D)
Harel-Katz et al. (2020) Effect size a	Community integration	39	IGPPD (RCT)	0.25	CVA	Israel	13 weeks (150 min)/weekly	Community integration (RNLI)
Harrison-Felix et al. (2018) Effect size a Effect size b	Social skills training	179	IGPPD (RCT)	− 0.26 0.01	TBI	USA	13 weeks (90 min)/weekly	Emotional distress (BSI-18) Quality of life (SWLS)
Horne et al. (2019) Effect size a	Neurorehabilitation psychology	47	IGPPD (RCT)	0.01	CVA	UK	11 weeks (120 min)/weekly	Emotional distress (GHQ-30)
Ingebretsen et al. (2023) Effect size a	Social skills training	49	IGPPD (RCT)	0.29	TBI (13), CVA (22), Other (14)	Norway	12 weeks (90 min)/weekly	Quality of life (PQOL)
Johansson et al. (2012)	Neurorehabilitation psychology	36	IGPPD (RCT)	n/a	TBI (15), CVA (21)	Sweden	8 weeks (150 min)/weekly	Anxiety (CPRS-A) Depression (CPRS-D)
Johansson et al. (2015) Effect size a Effect size b Effect size a Effect size b	Neurorehabilitation psychology	34	IGPPD (Controlled clinical trial)	− 0.18 0.24 0.44 0.48	TBI (16), CVA (18)	Sweden	8 weeks (150 min)/weekly	*Face-to-face group* Anxiety (CPRS-A) Depression (CPRS-D) *Internet group* Anxiety (CPRS-A) Depression (CPRS-D)
Joo et al. (2010) Effect size a Effect size b Effect size c	Neurorehabilitation psychology	11	SGPPD (Cohort)	0.60 0.72 0.76	CVA	Korea	8 weeks (150 min)/weekly	Anxiety (STAI-State) Anxiety (STAI-Trait) Depression (BDI-II)
Keegan et al. (2024)	Social skills training	6	SGPPD	n/a	TBI	Australia	8 weeks (130 min)/weekly	Quality of life (SWLS) Community integration (PARTO-17)
Kjeldgaard et al. (2014) Effect size a Effect size b Effect size c Effect size d Effect size e Effect size f Effect size g Effect size h Effect size i	Neurorehabilitation psychology	72	IGPPD (RCT)	− 0.04 − 0.30 − 0.41 0.17 − 0.06 0.09 − 0.06 0.02 0.01	Mild TBI	Denmark	9 weeks (120 min)/weekly	Quality of life (SF-36–Physical) Quality of life (SF-36–Physical) Quality of life (SF-36–Pain) Quality of life (SF-36–Health) Quality of life (SF-36–Fatigue) Quality of life (SF-36–Social) Quality of life (SF-36–Psychological) Quality of life (SF-36–Psychological) Emotional distress (SCL-90-R)
Kongkasuwan et al. (2016) Effect size a Effect size b Effect size c	Activity-based	118	IGPPD (RCT)	0.70 0.13 0.54	CVA	Thailand	4 weeks (90–120 min)/2 × per week	Depression (HADS-D) Anxiety (HADS-A) Quality of life (PTQL)
Künzi et al. (2022) Effect size a	Neurorehabilitation psychology	31	IGPPD (RCT)	− 0.17	Trauma (10), non-trauma (21)	Switzerland	6 weeks (75 min)/2 × per week	Emotional distress (BSI-18)
Lee et al. (2014) Effect size a Effect size b	Activity-based	21	IGPPD (Cohort analytic)	− 0.65 − 0.64	TBI	USA	8 weeks (60 min)/2 × per week	Depression (BDI-II) Quality of Life (Life-3)
Lee et al. (2017)	Community integration	17	IGPPD (Cohort analytic)	n/a	CVA	USA	3 weeks (90 min)/2 × per week	Community integration (RNLI) Quality of life (SIS)
Liu et al. (2023) Effect size a Effect size b Effect size c	Neurorehabilitation psychology	140	IGPPD (RCT)	2.14 0.02 1.17	CVA	China	4 weeks (45–60 min)/1–2 × per week	Depression (HDRS) Quality of Life (SF-12–Physical) Quality of Life (SF-12–Psychological)
Majumdar and Morris (2019) Effect size a Effect size b Effect size c Effect size d	Neurorehabilitation psychology	53	IGPPD (RCT)	0.37 0.31 − 0.03 0.41	CVA	UK	4 weeks (120 min)/weekly	Depression (PHQ-9) Anxiety (GAD-7) Quality of life (EQ-5D-5L) Emotional distress (WEMWBS)
McCarron et al. (2019) Effect size a Effect size b Effect size c Effect size d	Social skills training	10	SGPPD (Cohort)	0.58 1.23 0.67 0.26	TBI	USA	52 weeks (90 min)/weekly	Quality of life (QOLIBRI–Social) Quality of life (QOLIBRI–Psychological) Depression (BDI-II) Anxiety (BAI)
Mitchell et al. (2014)	Activity-based	12	SGPPD (Cohort)	n/a	Trauma (10), non-trauma (2)	Australia	1 × week-long intensive residential program	Quality of life (WHOQOL)
Moustgaard et al. (2007) Effect size a Effect size b Effect size c Effect size d Effect size e Effect size f Effect size g	Neurorehabilitation psychology	23	SGPPD (Cohort)	0.71 1.17 1.18 0.75 1.18 0.72 0.93	CVA	Canada	9 weeks (105 min)/weekly	Anxiety (BAI) Depression (BDI-II) Anxiety (HADS-A) Depression (HADS-D) Quality of life (SF-36–Physical) Quality of life (SF-36–Psychological) Quality of life (SS-QOL)
Muenchberger et al. (2011)	Community integration	52	SGPPD (Cohort)	n/a	TBI (20), CVA (17), other (1)	Australia	6 weeks (not reported)/ weekly	Emotional distress (DASS-21) Social support (MSPSS)
Nayak et al. (2000) Effect size a	Activity-based	18	IGPPD (Cohort analytic)	0.26	TBI, CVA	USA	Not reported (not reported)/ 2–3 × per week (max of 10 sessions)	Depression (Faces Scale)
Niu et al. (2022) Effect size a	Neurorehabilitation psychology	104	IGPPD (RCT)	0.12	CVA	China	Not reported (45–55 min)/5 sessions	Depression (HDRS)
Ownsworth et al. (2000)	Neurorehabilitation psychology	21	SGPPD (Cohort)	n/a	TBI (16), CVA (3), other (2)	Australia	16 weeks (180 min)/weekly	Quality of life (SIP)
Ownsworth et al. (2008) Effect size a Effect size b Effect size c	Neurorehabilitation psychology	11	IGPPD (RCT)	0.20 0.23 0.73	TBI, CVA, other	Australia	8 weeks (90 min)/weekly	Community integration (BICRO-39–Social) Community integration (BICRO-39–Productivity) Community integration (BICRO −39–Psychological)
Patchwood (2024) Effect size a Effect size b Effect size c	Neurorehabilitation psychology	17	IGPPD (Cohort analytic)	0.42 0.80 − 0.23	CVA	UK	9 weeks (not reported)/weekly	Depression (HADS-D) Anxiety (HADS-A) Quality of Life (EQ-5D-5L)
Raghavan et al. (2016)	Activity-based	13	SGPPD (Cohort)	n/a	CVA	USA	6 weeks (45 min)/2 × per week	Quality of life (SIS) Quality of life (WHO-5)
Sinnakaruppan et al. (2005) Effect size a Effect size b Effect size c Effect size d Effect size e Effect size f	Neurorehabilitation psychology	41	IGPPD (Controlled clinical trial)	0.22 0.18 0.34 0.43 0.23 0.43	TBI	UK	8 sessions (150 min)/(not reported)	Depression (HADS-D) Anxiety (HADS-A) Emotional distress (GHQ-28–Somatic) Anxiety (GHQ-28–Anxiety) Emotional distress (GHQ-28–Social dysfunction) Depression (GHQ-28–Depression)
Stubberud et al. (2019) Effect size a Effect size b Effect size c	Neurorehabilitation psychology	8	SGPPD (Cohort)	0.33 0.21 0.35	TBI (3), CVA (5)	Norway	4 weeks (180 min)/not reported	Emotional distress (HADS) Depression (HADS-D) Anxiety (HADS-A)
Tamplin et al. (2013) Effect size a Effect size b Effect size c Effect size d Effect size e Effect size f	Activity-based	13	SGPPD (Cohort)	− 0.31 0.24 0.19 − 0.05 0.54 0.39	CVA	Australia	20 weeks (120 min)/weekly	Quality of life (SIS–Cognition) Quality of life (SIS–Communication) Quality of life (SIS–Global) Social support (SOBI–Psychological) Social support (SOBI–Antecedent) Emotional distress (GHQ-12)
Tarrant et al. (2021) Effect size a Effect size b Effect size c	Activity-based	41	IGPPD (RCT)	− 0.16 − 0.12 0.08	CVA	UK	10 weeks (90 min)/weekly	Quality of life (SAQOL-39) Quality of life (EQ-5D-5L) Community integration (RNLI)
Tham et al. (2023) Effect size a Effect size b	Neurorehabilitation psychology	27	SGPPD (Cohort)	0.31 0.15	CVA	Singapore	3–4 weeks (60–90 min)/2–3 × per week	Depression (DASS-21-D) Anxiety (DASS-21-A)
Thomas (2004) Effect size a	Activity-based	22	IGPPD (Cohort analytic)	0.30	TBI (18), CVA (3), other (1)	Australia	9-day program + 3–4 months of fortnightly meetings	Quality of life (QOLI)
Tornås et al. (2016) Effect size a Effect size b Effect size c Effect size d	Neurorehabilitation psychology	70	IGPPD (RCT)	0.07 0.22 − 0.08 0.14	TBI (45), CVA (15), other (10)	Norway	8 weeks (120 min)/fortnightly	Emotional distress (HSCL-25) Depression (HSCL-25-D) Anxiety (HSCL-25-A) Quality of life (QOLIBRI)
Tsaousides et al. (2014)	Neurorehabilitation psychology	7	IGPPD (Cohort)	n/a	TBI	USA	8 weeks (60 min)/2 × per week	Emotional distress (DERS) Emotional distress (BREQ)
Tsaousides et al. (2017) Effect size a Effect size b	Neurorehabilitation psychology	91	IGPPD (Cohort)	0.40 0.40	TBI	USA	12 weeks (60 min)/2 × per week	Emotional distress (DERS) Quality of life (SWLS)
Visser et al. (2016)	Neurorehabilitation psychology	166	IGPPD (RCT)	n/a	CVA	Netherlands	8 weeks (90 min)/weekly	Quality of life (EQ-5D-5L) Depression (CES-D) Quality of life (SS-QoL)
Walker et al. (2005) Effect size a Effect size b	Activity-based	11	SGPPD (Cohort)	0.35 0.11	TBI	Australia	9 × monthly meetings + 9-day program + 4 months of fortnightly meetings	Depression (DASS-21-D) Anxiety (DASS-21-A)
Wang (2020) Effect size a Effect size b	Neurorehabilitation psychology	134	IGPPD (RCT)	0.37 0.42	CVA	China	8 weeks (120 min)/weekly	Depression (CES-D) Quality of life (FACT-Br)
Ward (2016) Effect size a Effect size b Effect size c	Neurorehabilitation psychology	48	SGPPD (Cohort)	0.60 0.39 0.26	CVA	Australia	7–10 weeks (120–180 min)/weekly	Depression (BDI-II) Depression (HADS-D) Anxiety (HADS-A)

*SGPPD* single-group pre-post design, *IGPPD* independent group pre-post design, *TBI* traumatic brain injury, *CVA* cerebral vascular accident.

To calculate each effect size, we extracted means, standard deviations, correlations between the scores pre and post intervention, and sample size. Following recommendations from Borenstein, Morris, and colleagues (Borenstein et al., 2021; Morris, 2008; Morris & DeShon, 2002), we calculated a common effect size metric in the form of standardised mean difference to allow for comparable effect size estimations across studies. For designs employing pre-and post measures, we used the pooled pre-intervention standard deviation as the denominator for the effect size calculation because this is not influenced by the intervention and provides more accurate estimations that are more comparable across studies (Morris, 2008). For independent-groups pre-post intervention designs, we used the following formula (Morris, 2008):

$$d_{IGPP}=\frac{\left(M_{post,\;E}-M_{pre,\;E}\right)-\left(M_{post,\;C}-M_{pre,\;C}\right)}{SD_{pre,\;P}}$$

where *M*_*post*_ is the sample mean post-intervention and *M*_*pre*_ is the sample mean pre-intervention (for the treatment and control group) and *SD*_*pre,P*_ is the pooled pre-intervention standard deviation. For single-group pre-post intervention designs, we used the following formula (Morris & DeShon, 2002):

$$d_{SGPP}=\frac{\begin{array}{c}{}\;M_{post_{,\;}E}-M_{pre_{,\;}E}\end{array}}{SD_{pre,\;E}}$$

where *SD*_*pre,E*_ is the pre-intervention standard deviation. Finally, to account for the positive bias of Cohen’s *d*, we applied Hedges’ correction factor to each effect size using the following formula (Hedges, 1981):

$$J=1-\frac3{4df-1}$$

where *df* is the degrees of freedom.

### Statistical Analysis

We conducted a three-level random-effects meta-analysis to calculate the summary standardised mean effect size, including 95% confidence intervals around the mean across the studies. This model accounts for three sources of variance: sampling variance of the observed effect sizes (*Level 1*), variance between effect sizes from the same study (within-study variance; *Level 2*), and the variance between studies (*Level 3*) (Cheung, 2014; Van den Noortgate et al., 2015). The three-level approach to meta-analysis allows for the modelling of dependent effect sizes extracted from the same study without requiring estimates of the correlations between those effect sizes (Moeyaert et al., 2017). This approach enables analyses to include all available effect sizes, preserving all information and maximising statistical power. We conducted analyses with RStudio (version 4.3.3; R Core Team, 2024) using the metafor package (Viechtbauer, 2010). We calculated the multilevel version of *I*^*2*^ following Cheung's (2014) formula which estimates the proportion of total variance that is associated with each of the three levels. To calculate the proportion of total variance that is associated with each of the three levels, we followed Cheung’s (2014) multilevel version *I*^2^ formula. This formula splits the heterogeneity into two parts: one attributable to true effect size differences within clusters, and the other to between-cluster variation.

All model parameters were estimated using the restricted maximum likelihood estimation method. We also used an 80% prediction interval around the mean effect size (indicating the 80% likelihood of the range of the effect size of a new study; Raudenbush, 2009).

We conducted additional moderated meta-analyses to examine effect sizes between intervention types and outcomes. The four types of interventions included neurorehabilitation, psychology, activity-based, social skills training, and community integration. The six outcomes included depression, anxiety, emotional distress, quality of life, community integration, and social support.

### Bias Analysis

Potential publication bias was examined using funnel plot (Egger et al., 1997), Egger’s test, and re-running the meta-analysis with sample size as a moderator (Kühberger et al., 2014). Funnel plot visualisations have not been developed for three-level meta-analyses, and so multiple dependent effect sizes within a study were averaged before plotting the effect size against standard error (in the funnel plot provided).

In addition, a risk of bias analysis for each study was conducted independently by two authors (HM and KT) using the Effective Public Health Practice Project Quality Assessment Tool for Quantitative Studies (EPHPP, 2009). Studies were reviewed for selection bias, study design, confounders, blinding, data collection methods, withdrawals, and drop-outs, with a global rating of ‘strong’, ‘moderate’ or ‘weak’ provided.

## Results

### Study Selection

**Fig. 2:**
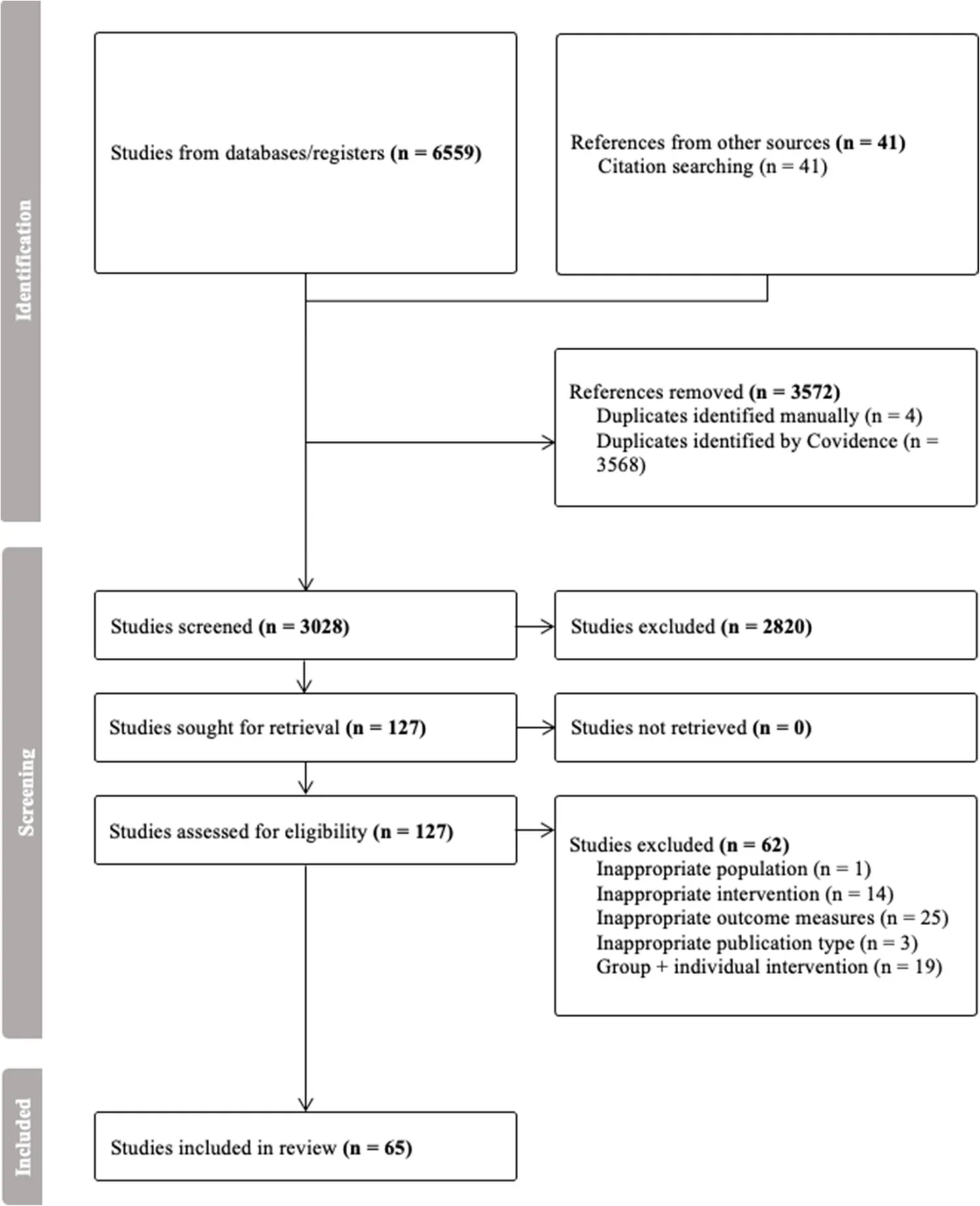
PRISMA flow chart for selected studies

The results of the systematic literature search strategy are presented in Fig. 2. The first author (HM) screened all 3028 titles and abstracts (after 3572 duplicates were excluded) for eligibility using Covidence. Full-text review of the remaining 127 studies was also conducted in Covidence, independently by two authors (HM and KT) with 98% agreement (*κ* = 0.88, 95% CI, 0.80–0.96), indicating strong inter-reliability. The full-text screening yielded a total of 65 records that fulfilled the inclusion criteria. Forty-eight of these included sufficient data and were included in the meta-analysis.

### Study Characteristics

Details of all studies are provided in Table 1. The 65 studies reported on a total of 3529 patients. The sample size of eligible studies varied from 6 to 705, with a median of 30 and a mean sample size of 54. The studies were conducted in 17 different countries across 4 continents: 23 from North America (USA, Canada), 19 from Europe (UK, Netherlands, Norway, Denmark, Sweden, Italy, Spain, and Switzerland), 7 from Asia (China, Israel, Thailand, Korea, and Singapore), and 16 from Oceania (Australia, New Zealand).

### Participants

There was a relatively equal distribution of ABI aetiology with 20 traumatic brain injury only studies, 23 stroke only studies, and 22 studies with mixed aetiologies including stroke, TBI, hypoxia, tumour, infection, and neurotoxicity. Sex distribution was relatively equal with 49% of the overall sample recorded as female. The age of participants ranged from 18 to 94 years with the median age 47 (mean = 49). Injury severity ranged from ‘mild’ to ‘extremely severe’, both within and between studies, although 28 studies did not report severity at all. Among those that did, definitions were rarely provided and varied considerably. Some studies provided anecdotal descriptions of severity (*n* = 12), some used functional or cognitive screening tools as a measure of severity (*n* = 7), and others used standardised clinical gradings such as length of post-traumatic amnesia (PTA) for TBI, the Glasgow coma scale (GCS) for mixed ABI, or the National Institutes of Health Stroke Scale (NIHSS) (*n* = 18). However, even among studies using standardised tools, classification criteria were inconsistently applied and most did not specify which severity classification system they followed. For instance, some studies adopted early PTA-based frameworks in which > 28 days denotes ‘extremely severe’ injury, whereas others classified ‘severe’ injury as PTA > 7 days (Ponsford et al., 2013). Time since injury onset also varied markedly, ranging from less than a week to 36 years.

### Experimental Design and Methodology

Using the study definition of psychosocial intervention type, there were 39 interventions classified as neurorehabilitation psychology, 14 as activity-based, 8 as social skills training, and 4 as community integration. Among the studies reviewed, 25 (38%) were cohort studies (i.e. one group that was evaluated pre + post-intervention), 24 (36%) were Randomised Controlled Trials (RCT), 11 (18%) were cohort analytic (i.e. observational study design of two groups compared pre + post intervention), and 5 (8%) were non-randomised controlled clinical trials. The distribution of RCT designs across the different intervention types varied with 16/39 (41%) RCTs among the psychological, 4/14 (29%) among the activity-based, 4/8 (50%) among social skills, and 1/4 (25%) in the community integration studies.

#### Intervention Design

The interventions varied markedly in methodology, even within the four categories. They were delivered in inpatient, outpatient, and community settings by a diverse range of professionals, including allied health practitioners (i.e. psychologists, neuropsychologists, speech pathologists, social workers, occupational therapists, and physiotherapists), nurses, specialist trainers (i.e. professional facilitators, yoga instructors, MBSR instructors, music therapists, and art therapists), and/or peer facilitators. While the facilitator’s role is an important factor, all facilitators in the reviewed studies were trained professionals, and most interventions incorporated multiple types of facilitators. This variability limits our ability to analyse or draw definitive conclusions about the specific impact of facilitator type on outcomes.

The duration of interventions ranged from 1 week to 12 months. Session length ranged from 45 min to 3 h. Most interventions (39 studies) ran weekly, though there was substantial variability in the remaining studies where the intensity of delivery ranged from daily delivery (4 studies), 2–3 times per week (18 studies), and fortnightly (1 study). Three studies did not report duration or intensity information. Group size ranged from 2 participants (5 studies) to 20 participants (1 study), while the average group size was 6 participants. Most groups were delivered in-person, although one study delivered half of their sessions as telephone-based group classes (Bay & Chan, 2019), and six studies delivered their group intervention online (Johansson et al., 2015; Keegan et al., 2024; Patchwood et al., 2024; Tham et al., 2023; Tsaousides et al., 2014, 2017). Forty-two of the studies included a comparison group in the analysis. Forty-one studies measured outcomes at baseline and then follow-up, with the latter ranging from 4 weeks to 2 years post-intervention.

#### Outcome Measures

A total of 51 different outcome measures were used to assess the 6 elements of psychosocial functioning that formed the focus of this review. Among the outcome measures, four scales focused on anxiety alone, eight on depression alone, and thirteen scales measured emotional distress. There were sixteen different scales measuring quality of life, three scales measuring social support, and seven scales measuring community integration. The Hospital Anxiety and Depression Scale (HADS) was the most frequently used measure of anxiety (used in 16 studies) and depression (used in 15 studies). The Brief Symptom Inventory (BSI-18) was the most frequently used measure of emotional distress, used in four studies. The Quality of Life after Brain Injury (QOLIBRI) and the 36-Item Short Form Health Survey (SF-36) were the most frequently used measures of quality of life, used in seven studies each. The Community Integration Questionnaire (CIQ) was the most frequently used measure of community integration, used in four studies. Measures of social support differed across the three studies using this index (i.e. Medical Outcomes Study Social Support Survey, Multidimensional Scale of Perceived Social Support, and Sense of Belonging Instrument).

#### Group Processes

Seventy percent of the studies (46/65) explicitly noted benefits associated with group delivery in intervention outcomes. These benefits included cost effectiveness (e.g. Aboulafia-Brakha et al., 2013; Bédard et al., 2014; Majumdar & Morris, 2019; Niu et al., 2022), peer learning (e.g. Anson & Ponsford, 2006b; Appleton et al., 2011; Chalmers et al., 2019; Harrison-Felix et al., 2018; Ingebretsen et al., 2023; Johansson et al., 2015), reduced social isolation and loneliness (Bédard et al., 2003; Fleming et al., 2009; Ward et al., 2016), increased self-awareness and self-efficacy (e.g. Backhaus et al., 2010, 2016; Gurr, 2009; Kjeldgaard et al., 2014) and promoting treatment group identity (Tarrant et al., 2021). There was also acknowledgement of the contribution that group processes of cohesion (Cuberos-Urbano et al., 2018; Tham et al., 2023), social support (Appleton et al., 2011; Blake et al., 2024; Cassel et al., 2024; Cuberos-Urbano et al., 2018; Donnelly et al., 2017; Gurr, 2009), and group fit or belonging (Ellis-Hill et al., 2019; Raghavan et al., 2016; Tamplin et al., 2013) made to outcomes.

Despite this positive narrative recognising the benefits of the group modality in delivery, only two studies explicitly assessed group process, and this was conducted using two different measures: the Group Climate Questionnaire (Backhaus et al., 2016) and the Social Identification Self-report Scale (Ellis-Hill et al., 2019). The Group Climate Questionnaire was used to assess levels of engagement with the intervention and found no significant differences between experimental and control groups. The Social Identification Self-Report Scale median scores were reported showing that identification increased across sessions and remained high in the final session. Neither study explored the impact of these group processes on intervention outcome. Therefore, no psychosocial group interventions in this review satisfactorily investigated group processes in their design.

### Quality Assessment

**Fig. 3:**
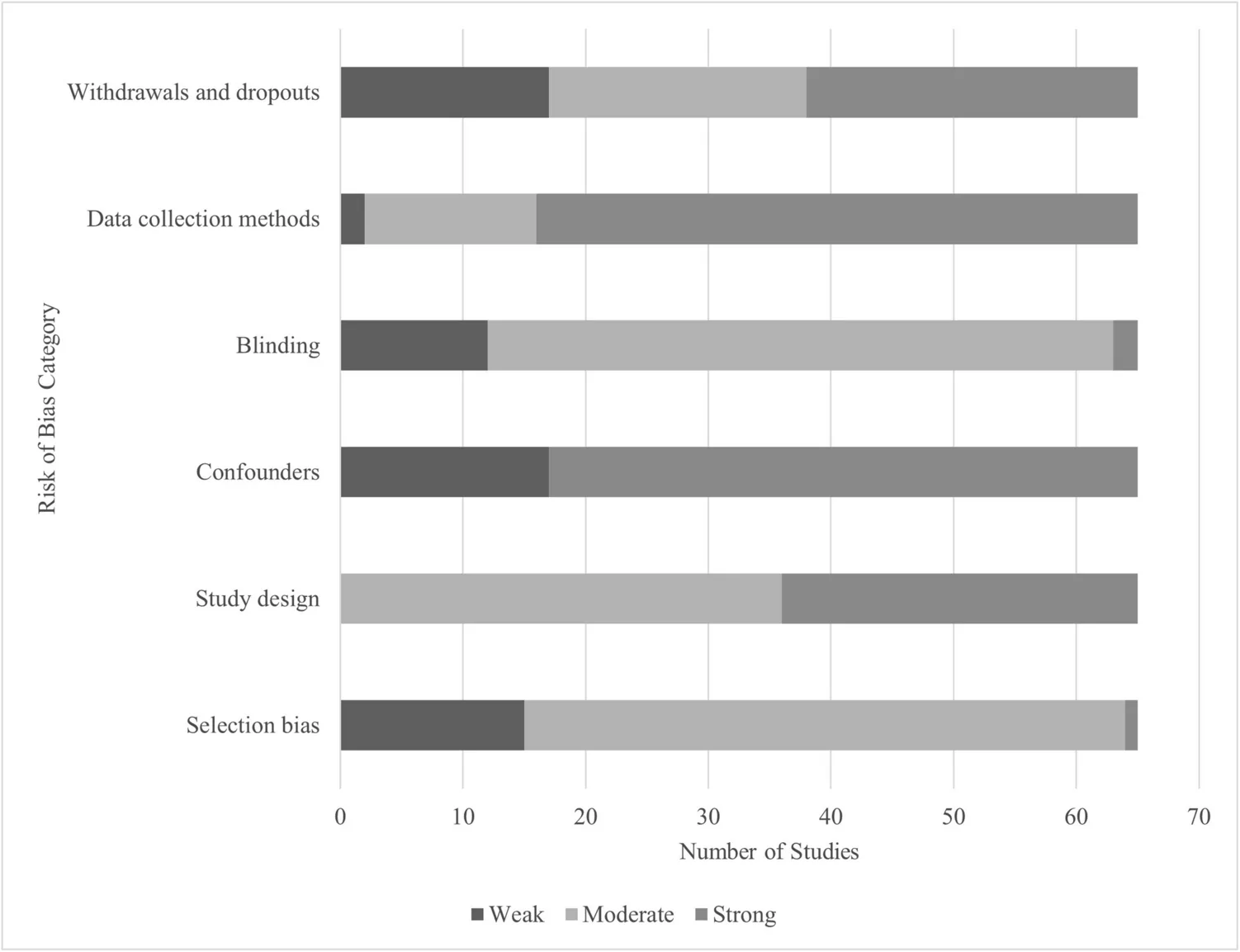
Methodological quality ratings by subcategories

Inter-rater agreement for the methodological quality assessment was moderate (*κ* = 0.67; 95% CI, 0.51–0.83). Of the 65 studies eligible for inclusion, the overall methodological quality of 22 studies was rated ‘strong’, 27 were ‘moderate’, and 16 were ‘weak’ (see Fig. 3 for an overview and Online Resource 3 for individual study ratings). The studies were difficult to rate accurately due to studies inadequately reporting on quality criteria, resulting in many “can’t tell” ratings. Most studies were rated highly for data collection methods as they used valid and reliable outcome measures. No study incorporated double-blinding, but this limitation is difficult to circumvent in rehabilitation interventions (Cicerone et al., 2019). Only one study (Donnelly et al., 2021) achieved a strong rating for selection bias as most of the studies included convenience samples from local hospitals or community groups. There was no discernible difference in methodological ratings between intervention types.

### Synthesis of Results

#### Neurorehabilitation Psychology Interventions

Among the neurorehabilitation psychology interventions, cognitive behavioural therapy (CBT), adapted for ABI populations, was the most widely used framework, applied in eleven studies (Aboulafia-Brakha et al., 2013; Anson & Ponsford, 2006a, 2006b; Arundine et al., 2012; Backhaus et al., 2010, 2016; Bradbury et al., 2008; Kjeldgaard et al., 2014; Tsaousides et al., 2014, 2017; Ward et al., 2016). These studies integrated rehabilitative and cognitive techniques to target anger management, adjustment and coping, emotional regulation and emotional distress, which are important areas of psychosocial difficulty faced in ABI populations. Additionally, three studies extended the CBT framework by incorporating goal management training (GMT), a metacognitive approach designed to support executive functioning through structured goal-setting and self-monitoring techniques (Cuberos-Urbano et al., 2018; Stubberud et al., 2019; Tornås et al., 2016). This integration highlights the adaptability of CBT to address specific cognitive deficits associated with ABI. Furthermore, one study adopted a more holistic approach by combining cognitive and psychological techniques, including CBT, to improve overall psychological wellbeing (Tham et al., 2023). Collectively, these studies highlight the prominence and versatility of CBT in neurorehabilitation, offering a robust foundation for addressing both emotional and cognitive rehabilitation needs in ABI populations.

Similarly, mindfulness-based interventions were prominent, with six studies employing mindfulness-based stress reduction program (MBSR; Azulay et al., 2013; Baldo et al., 2021; Bédard et al., 2003; Johansson et al., 2012, 2015; Joo et al., 2010) and two employing general mindfulness techniques (Bay & Chan, 2019; Combs et al., 2018). An innovative study integrated mindfulness and self-compassion with animal-assisted therapy (Künzi et al., 2022), reflecting a novel and creative application of mindfulness. Mindfulness-based cognitive therapy (MBCT), which combines CBT and mindfulness, was used in three studies (Bédard et al., 2014; Moustgaard et al., 2007; Wang et al., 2020), while acceptance and commitment therapy (ACT), integrating values-driven action and psychological flexibility with mindfulness strategies, was the focus of five studies (Blake et al., 2024; Liu et al., 2023; Majumdar & Morris, 2019; Niu et al., 2022; Patchwood et al., 2024). These approaches underscore the increasing recognition of mindfulness as a valuable component in managing emotional and psychosocial challenges in ABI rehabilitation.

Two studies focused specifically on enhancing problem-solving skills by promoting adaptive problem-solving attitudes and encouraging the implementation of effective coping behaviours to manage life stressors (Chalmers et al., 2019; Visser et al., 2016). These interventions emphasise the importance of equipping individuals with practical strategies to navigate the complexities of post-injury life. Holistic support group interventions were also a focus, integrating psychotherapeutic techniques, educational components, and cognitive rehabilitation to facilitate post-injury adjustment and coping (Horne et al., 2019; Ownsworth et al., 2000, 2008). These interventions integrated multiple therapeutic modalities aimed at improving self-awareness and psychosocial functioning, emphasising the value of group-based approaches in fostering interpersonal connection and self-reflection. Complementing this, another study employed a psychotherapeutic support group that encouraged participants to share their stroke and rehabilitation experiences, highlighting the therapeutic potential of peer support in promoting recovery and emotional adjustment (Gurr, 2009). Additionally, an educational training program was designed to address the cognitive and psychological changes associated with ABI, providing participants with the knowledge and tools to better understand and manage the impact of their condition (Sinnakaruppan et al., 2005). Collectively, these studies demonstrate the diverse methodologies used in ABI neurorehabilitation psychology, each targeting different facets of recovery to enhance well-being.

The studies predominantly measured depression (28/39), followed by anxiety (19/39), quality of life (18/39), emotional distress (14/39) and community integration (4/39) as outcomes. None of the neurorehabilitation psychology intervention studies assessed social support, indicating a potential gap in the focus of current research.

### Activity-Based Interventions

The category of activity-based intervention included a variety of programs designed to promote engagement and general well-being. Three studies used arts-based approaches, combining art-therapy with psychotherapy to address psychological needs in individuals with ABI (Di Vita et al., 2020; Ellis-Hill et al., 2019; Kongkasuwan et al., 2016). These interventions emphasised the therapeutic potential of creative expression as a means of coping and emotional processing. Music-based interventions were used in four studies, comprising two studies that assessed the effects of taking part in a choir (Tamplin et al., 2013; Tarrant et al., 2021), one study combining singing with instrument playing (Nayak et al., 2000), and another study integrating music with upper limb therapy (Raghavan et al., 2016). These interventions illustrate the dual focus of music-based approaches on enhancing emotional well-being and physical rehabilitation.

Two studies used yoga to support physical and emotional recovery (Donnelly et al., 2017, 2021). Leisure programs (i.e. organised activities, events, or initiatives) were the focus of three studies, two of which involved a 9-day Outward Bound adventure course aimed at fostering resilience and self-efficacy (Thomas, 2004; Walker et al., 2005), and one study aimed to increase participation in recreational social activities (Mitchell et al., 2014). Additionally, two studies integrated exercise with broader psychosocial goals, with one combining exercise with education on healthy behaviour change, including stress management (Faulkner et al., 2015), while the other paired exercise with a self-affirmation intervention (Lee et al., 2014).

Quality of life was the most frequently measured outcome across these interventions (12/14), highlighting its centrality in activity-based interventions for ABI rehabilitation. Depression, anxiety, and emotional distress were also assessed (7/14, 5/14, and 3/14 respectively), reflecting the psychological focus of these approaches. However, community integration and social supportfd, which are critical elements of post-injury recovery, were evaluated in only one study each, suggesting potential areas for further exploration. Overall, these interventions demonstrate the potential of group activity-based approaches to enhance both psychological and physical aspects of recovery in ABI rehabilitation. However, there are only a few studies for each activity-based approach, emphasising the need for further research in this area and a broader evaluation of potential psychosocial outcomes.

### Social Skill Training

The review identified eight social skill group interventions designed to address the unique interpersonal challenges that can impact social functioning in individuals with ABI. One intervention was adapted from the Improving First Impressions program (McDonald et al., 2008), focusing on increasing prosocial behaviours and reducing antisocial behaviours (Appleton et al., 2011). Two programs aimed to enhance social communication skills by utilising group processes to foster appropriate interactions (Dahlberg et al., 2007; Keegan et al., 2024). Two programs aimed to improve overall social competence, addressing broader aspects of interpersonal functioning (Cassel et al., 2024; McCarron et al., 2019). Three studies used the Group Interactive Structured Treatment (GIST) program, a comprehensive approach to address the underlying cognitive, communicative, and emotional impairments after ABI (Braden et al., 2010; Harrison-Felix et al., 2018; Ingebretsen et al., 2023). The GIST program’s structured and interactive format highlights its adaptability in addressing multifaceted social skill deficits.

Quality of life was assessed in all eight studies, suggesting that social skills training programs aim to improve overall functioning and well-being post-injury. Other outcomes varied across studies, with anxiety, depression and community integration measured in three studies each, and emotional distress measured in one study. Notably, none of the studies included social support as an outcome measure, indicating an area for future research. Overall, there is a diversity in social skills group intervention for ABI populations, with this review highlighting the need for broader and more consistent assessment of psychosocial outcomes, particularly social support, which is critical for successful community reintegration and interpersonal recovery.

### Community Integration Programs

This review found four community integration group programs targeting psychosocial recovery in ABI rehabilitation. Two studies implemented the Improving Participation After Stroke Self-Management (IPASS) group program, which integrates an evidence-based chronic disease self-management framework with a stroke-specific focus. This program addresses key areas such as managing at home, engaging in community and work environments, and improving social participation (Harel-Katz et al., 2020; Lee et al., 2017). Another of the community integration programs titled ‘Personal Environments Enhancing Rehabilitation’ (PEER) group aimed to address environmental factors, empowering participants to navigate and optimise their personal environments while maximising available supports (Fleming et al., 2009). Finally, the ‘Skills to Enable People with brain injury and their communitieS’ (STEPS) program aimed to build both personal and interpersonal resources to enhance community integration and participation (Muenchberger et al., 2011).

Outcome measures varied across studies. Community integration was the most frequently assessed outcome (three studies), followed by social support (two studies). Anxiety, depression, emotional distress, and quality of life were each measured in only one study. These findings highlight both the paucity and diversity in current group-based approaches to community integration for ABI populations. Additionally, the limited and inconsistent measurement of critical variables, such as psychological well-being and social support, indicates a need for more comprehensive and standardised evaluation in future research.

## Meta-analysis

### Focal Analysis

**Fig. 4:**
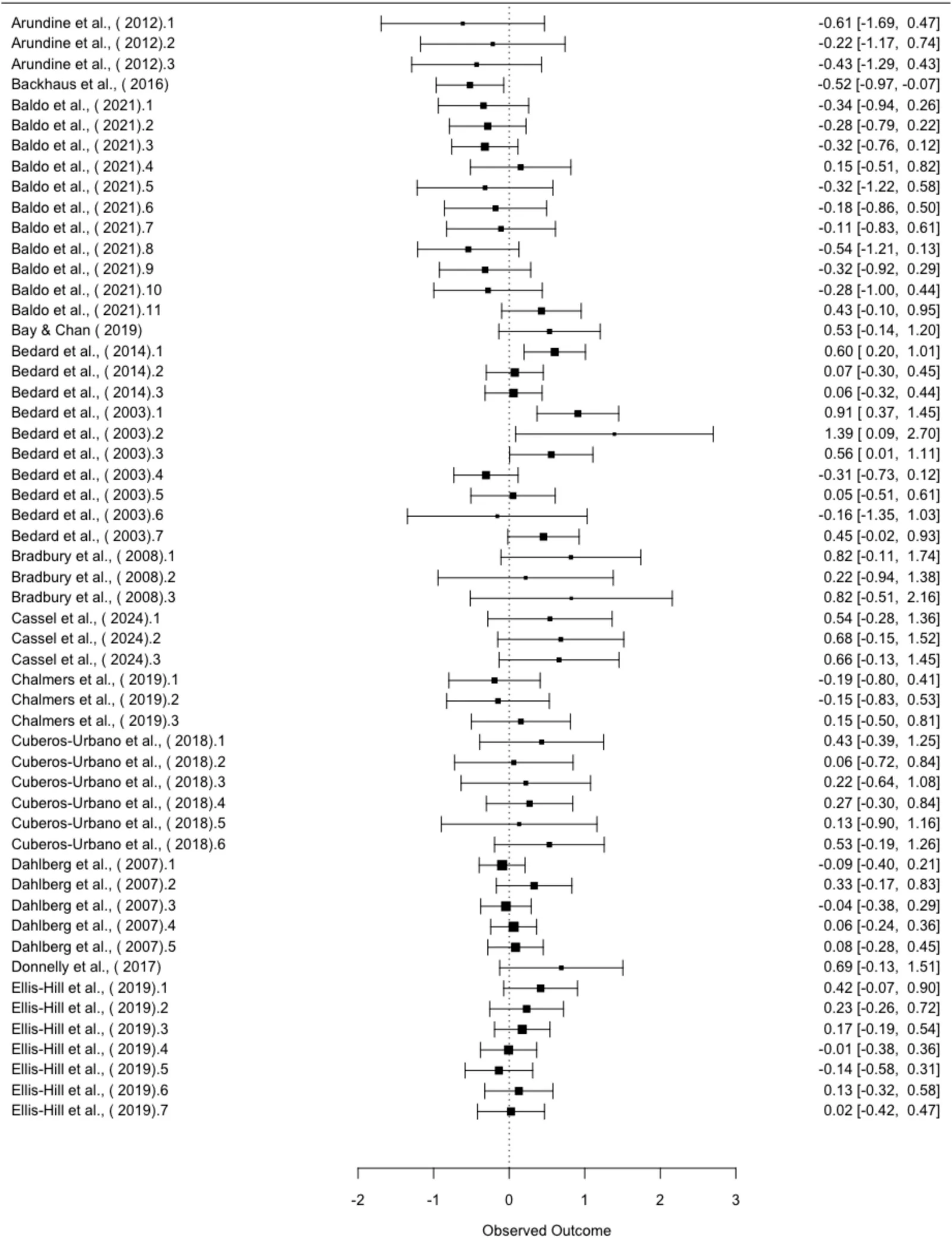

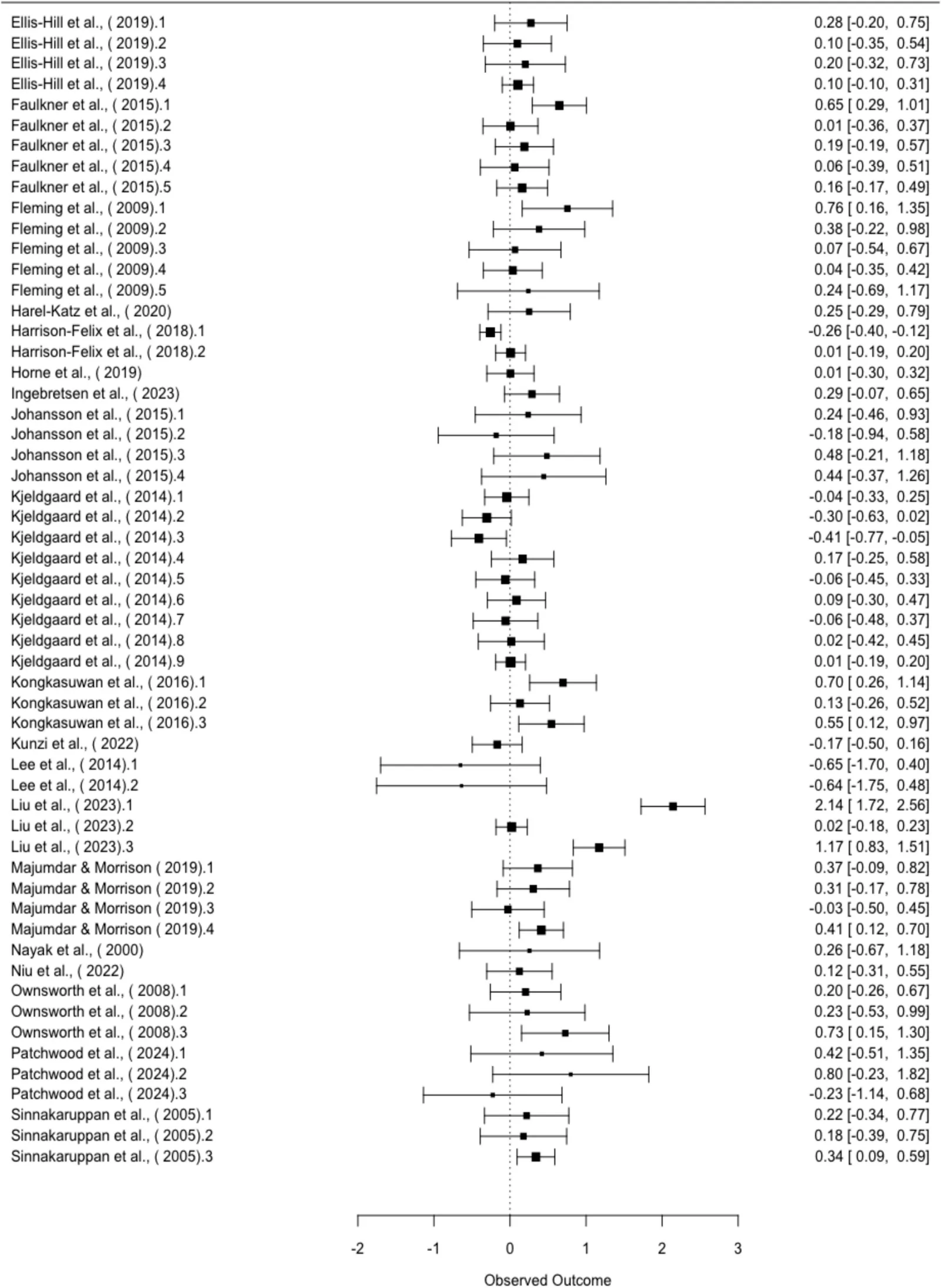

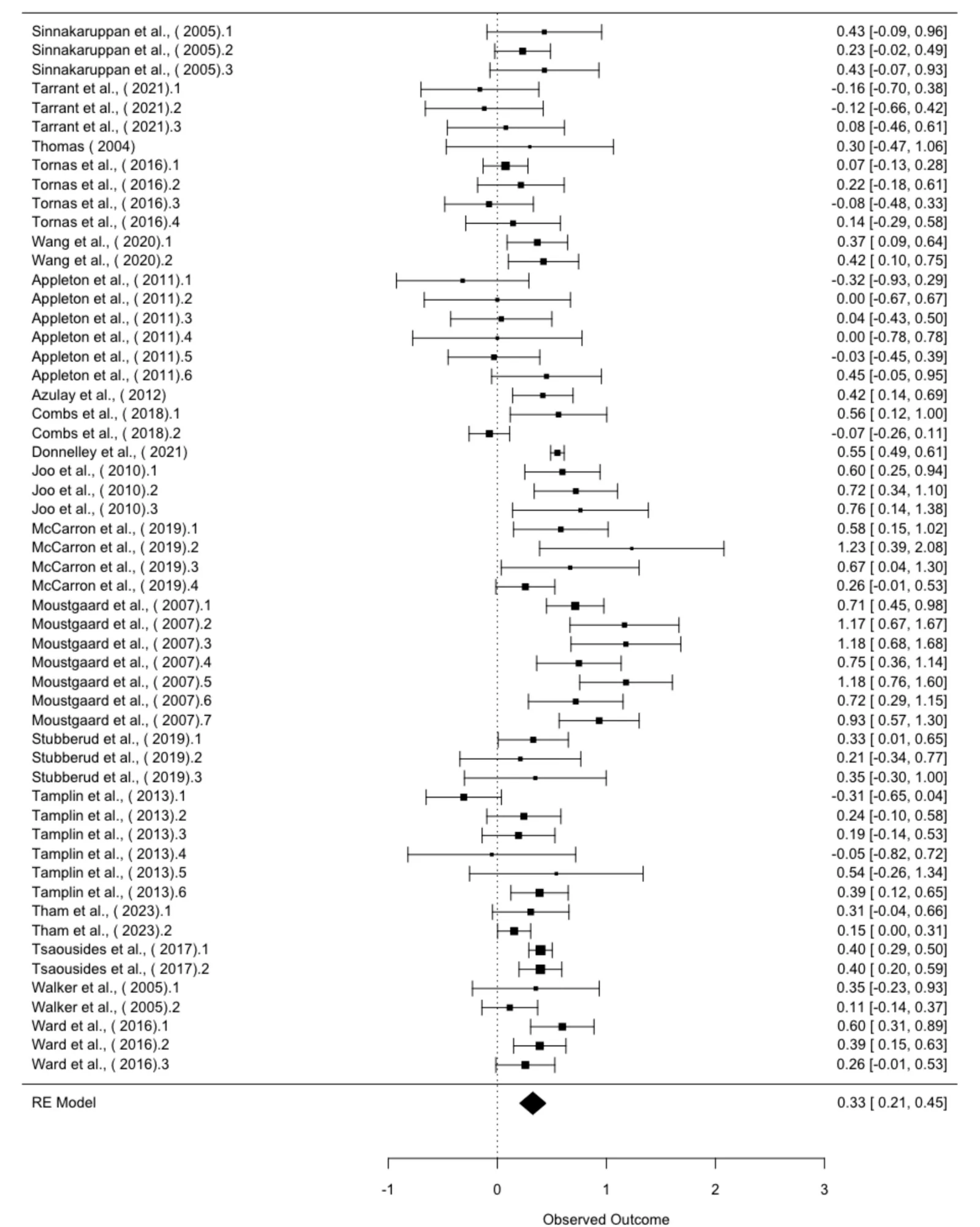
Forest plot of effect sizes for psychosocial intervention outcomes

The total sample for meta-analysis included data from 48 manuscripts published between 2000 and 2024, describing 165 effect sizes derived from a total sample size of 2653 individuals with ABI. An overview of the effects of each study is presented in Table 1 and Fig. 4. The overall effect indicates that group psychosocial interventions had a significant small positive effect on psychosocial outcomes, Hedges’ *g* = 0.24, 95% CIs [0.16, 0.33], *t*(164) = 5.53, *p* < 0.001. A larger amount of the total variance (*Q*(164) = 607.57, *p* < 0.001, τ^2^ = 0.09) was due to differences between studies (*Level 3*:* I*^2^ = 44.52%), rather than within studies (*Level 2*: *I*^2^ = 29.38%) and sampling error (*Level 1*:* I*^2^ = 26.09%). The 80% prediction interval (Raudenbush, 2009) indicated an 80% chance that the effect size of a new study will fall within the range of a standardised mean effect size (Hedges’ *g*) of − 0.15 and 0.63.

We conducted moderation analyses to explore whether demographic and injury-related factors influenced the results. The analyses revealed that age was not a statistically significant moderator (*F* (1,162) = 0.86, *p* = 0.354), and neither was sex (*F* (1,163) = 0.01, *p* = 0.939). In terms of injury aetiology, studies were categorised into three groups: stroke only (*k* = 18), traumatic brain injury only (*k* = 13), and mixed aetiology (*k* = 17). However, aetiology was not a significant moderator of the results (*F* (2,162) = 0.65, *p* = 0.521).

### Efficacy of Intervention as a Function of Type

Table 2 displays the results of the three-level meta-analysis examining intervention type as a moderator, which was non-significant (*F* (3, 161) = 0.46, *p* = 0.707). A larger amount of the total variance (*Q*(161) = 537.12, *p* < 0.001, *τ*^2^ = 0.09) was due to differences between-studies (*Level 3*: *I*^2^ = 45.57%), than within-studies (*Level 2*:* I*^2^ = 28.96%) and sampling error (*Level 1*:* I*^2^ = 25.47%)*.* The moderation results show that no single intervention was more effective than others in improving psychosocial outcomes. Nevertheless, only the neurorehabilitation psychology interventions demonstrated a significant positive effect on outcomes (Hedges’ *g* = 0.28, 95% CI [0.17, 0.39]), while the remaining interventions were non-significant.

**Table 2:** Results of the effectiveness of group psychosocial interventions as a function of intervention type

Intervention type	*k*	*ES*	*N*	Hedges’ *g*	95% CI	*t*	*p*	80% prediction interval
Activity based	9	34	352	0.15	[− 0.06, 0.36]	1.38	0.169	− 0.27, 0.56
Community integration	2	6	75	0.27	[− 0.20, 0.73]	1.12	0.266	− 0.23, 0.76
Neurorehabilitation psychology	31	104	1908	0.28	[0.17, 0.39]	5.03	0.000	− 0.12, 0.68
Social skills training	6	21	318	0.19	[− 0.05, 0.43]	1.59	0.113	− 0.23, 0.62

### Efficacy as a Function of Psychosocial Outcome

Table 3 presents the results from the three-level meta-analysis examining psychosocial outcome type as a moderator, which was non-significant *F* (5, 159) = 1.99,* p* = 0.084. A larger amount of the total variance *Q*(159) = 551.76, *p* < 0.001, *τ*^2^ = 0.08 was attributed to differences between-studies (*Level 3*: *I*^2^ = 45.13%), than within-studies (*Level 2*: *I*^2^ = 27.67%) and sampling error (*Level 1*:* I*^2^ = 27.20%). The moderation results show that no specific psychosocial outcome measure demonstrated a significantly greater effect than others. However, community integration and social support had the fewest studies and the smallest sample sizes included in the analysis, which may explain why they were the only two outcomes that did not show significant effects.

**Table 3:** Results for the effectiveness of group psychosocial interventions as a function of psychosocial outcomes

Psychosocial outcome	*k*	*ES*	*N*	Hedges’ *g*	95% CI	*t*	*p*	80% prediction interval
Anxiety	22	26	709	0.19	[0.05, 0.34]	2.65	0.009	− 0.19, 0.58
Community integration	7	15	219	0.08	[− 0.14, 0.31]	0.73	0.467	− 0.32, 0.49
Depression	30	37	1213	0.39	[0.26, 0.52]	5.85	0.000	0.00, 0.77
Emotional distress	15	17	698	0.20	[0.05, 0.35]	2.64	0.009	− 0.19, 0.59
Quality of life	28	67	2051	0.23	[0.12, 0.34]	4.10	0.000	− 0.15, 0.62
Social support	2	3	49	0.26	[− 0.29, 0.82]	0.94	0.349	− 0.26, 0.78

### Bias Analysis

**Fig. 5:**
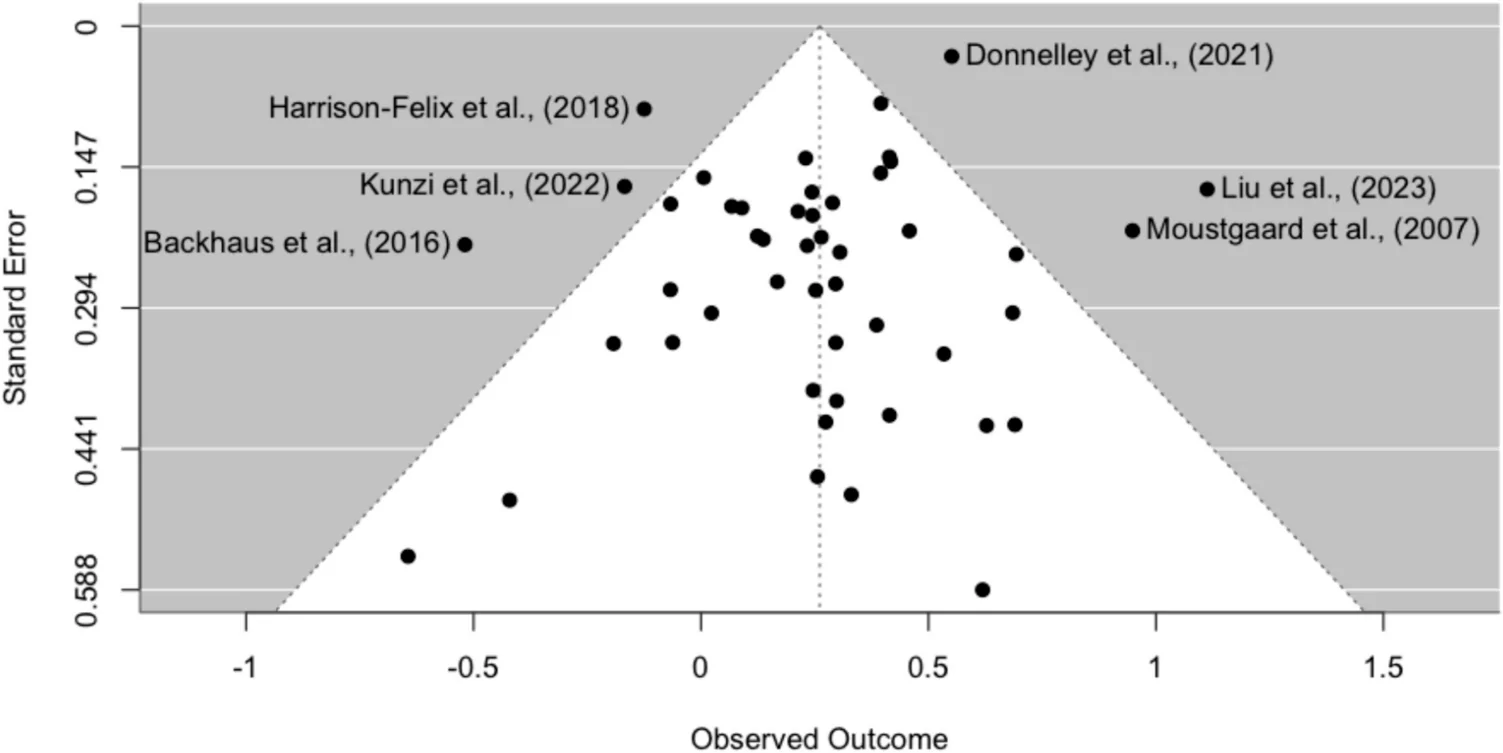
Funnel plot asymmetry analysis

Visual inspection of the funnel plot (see Fig. 5) revealed no clear indication of asymmetry. We followed this up with Egger and colleagues’ (1997) regression test, examining whether the effect size was predicted by an estimate’s standard error. The analysis indicated that the standard error was not a significant predictor of the effect size (*b* = 0.46, *t*(44) = − 1.86, *p* = 0.07), further suggesting no evidence of asymmetry.

To explore whether risk of bias ratings impacted the results, we conducted an additional analysis excluding the studies with a weak risk of bias rating. The resulting effect size estimate remained significant (Hedges *g* = 0.24, 95% CIs [0.12,0.36], *t*(114) = 3.99, *p* < 0.001) and was slightly higher than the estimate when these studies were included. Additionally, we performed a moderation analysis to examine whether the effect size estimate varied based on risk of bias rating. This analysis indicated risk of bias was not a significant moderator (*F* (2,162) = 2.53, *p* = 0.08). These findings demonstrate that excluding studies with weak risk of bias ratings does not substantially alter the overall results.

Finally, we examined whether the effect size could be predicted by a study’s sample size. Publication bias is typically indicated by a significant relationship between sample size and effect size, such that small studies tend to report relatively larger effects (Kühberger et al., 2014). Moderation by sample size yielded no evidence that the effect size was predicted by sample size (*F*(1, 163) = 1.09, *p* = 0.297), which provided further evidence that the results were not substantially affected by publication bias.

### Sensitivity Analysis

We assessed influential cases and outliers following the recommendations of Viechtbauer and Cheung (2010), using extensions of standard diagnostics for independent regression models. These extensions were necessary because the effect sizes in the current study were not independent. Specifically, cases were evaluated using (1) standardised residuals, (2) Cook’s distance and (3) DFBetas (Viechtbauer & Cheung, 2010). There were 2 cases with standardised residuals greater than 2.6. This is more than expected assuming a normal distribution with the current sample. Cases were considered influential if their Cook’s distance was larger than the median plus six times the interquartile range of Cook’s distance, and if DFBETA was greater than 0.178 (2/sqrt(*n*)). A total of eight studies had influence on the model’s estimate. Running the meta-analysis without these cases did not change the result of the overall model (Hedges’ *g* = 0.21–0.24) or the moderation results.

## Discussion

The present review aimed to identify the range of group-based psychosocial interventions used in ABI rehabilitation, evaluate their impact on psychosocial outcomes, and examine how group processes contribute to these outcomes. The narrative synthesis highlights the significant role of CBT in group-based neurorehabilitation psychology interventions as, consistent with previous reviews, it is the most widely used framework across studies, solidifying its prominence in ABI rehabilitation. Adapted CBT interventions effectively targeted psychosocial challenges and were often combined with other holistic psychological and cognitive techniques that underscore its adaptability in promoting emotional and cognitive recovery. Mindfulness approaches also featured as a prominent neurorehabilitation psychology approach, with a recent rise in the use of ACT-informed interventions. Other group-based psychosocial interventions including activity-based, social skills training, and community integration programs showcased the diversity and creativity of approaches to ABI rehabilitation in addressing psychosocial outcomes. However, the evidence behind these approaches remains limited, with few studies evaluating their efficacy and exploring broader psychosocial outcomes.

This is the first review to examine the nature and efficacy of *group* psychosocial interventions used in rehabilitation settings through a meta-analysis. To address the heterogeneity between the four categories of psychosocial interventions, a three-level meta-analysis was used. The findings indicated that group-based psychosocial interventions had a small but statistically significant positive effect on psychosocial outcomes in brain injury populations. This effect remained significant even after controlling for variables such as age, sex, and aetiology. While the inclusion of highly diverse studies provided a broader understanding of intervention impacts, it may have reduced the precision of the overall effect size estimate.

There was also variation in the effect size across intervention types. Consistent with the narrative synthesis, we found that neurorehabilitation psychology group interventions were the most frequently studied and moderation analysis revealed an independent significant effect on psychosocial outcomes (Hedges’ *g* = 0.28). This effect may be due to the number of studies included or is perhaps due to the neurorehabilitation psychology interventions being theoretically grounded in ways that informed their design. In contrast, activity-based, community integration, and social skills group interventions had non-significant effects on psychosocial outcomes. However, these findings may be driven in part by sample size, as there were more studies exploring neurorehabilitation psychology group interventions (*k* = 31) than the other intervention categories (*k* ranged from 2 to 9).

The results also showed that the magnitude of the effect of intervention varied across different psychosocial outcomes (from *g* = 0.08 to *g* = 0.39) with the smallest effect sizes found for community integration and social support, which again, is partially attributable to sample size as they were the least studied of the psychosocial constructs.

The third aim of this review was to examine how group processes contribute to group intervention outcomes. The narrative synthesis found that despite 70% of the included studies proclaiming the importance of group process on outcomes, only two studies measured group process, and neither study explored the impact of these processes on intervention outcome.

### Limitations and Recommendations for Future Research and Practice

Findings from the present study highlight the potential for psychosocial group interventions to improve outcomes in brain injury rehabilitation. However, several limitations warrant caution when interpreting these results. Variability in injury severity, aetiology, and time since injury both within and between the studies complicates the determination of which individuals benefit most from these interventions. Moreover, injury severity and mechanism were not consistently reported or clearly defined, with studies using varied classification methods and frameworks. This heterogeneity limited the ability to stratify findings based on injury characteristics beyond broad injury type. While moderation analysis revealed no significant differences in outcomes across studies involving stroke, TBI, and mixed injury groups, the overall methodological quality of included studies was mixed, underscoring the need for more rigorous research designs (particularly RCTs). This aligns with prior calls for higher-quality research in ABI rehabilitation (e.g. Cattelani et al., 2010; Cullen et al., 2007; Teasell et al., 2024). The focus on published and English-language literature may also weaken the conclusions drawn and mean that the current review is limited in generalisability.

Small sample sizes, heterogenous populations, and diverse outcome measures also limit the generalisability of the current findings. Furthermore, the lack of longitudinal follow-up assessments makes it challenging to attribute observed benefits directly to the intervention or evaluate their long-term efficacy. Additionally, our risk of bias analysis revealed insufficient reporting of key study characteristics, a common issue in similar intervention reviews (Kirby et al., 2017; Steffens et al., 2021). Ambiguity in the quality assessment tool itself further contributed to a moderate interrater agreement, potentially introducing bias and reducing the reliability of the results. To address these limitations, future research must prioritise improved reporting standards and adhere to guidelines such as CONSORT and PRISMA to enhance the transparency, replicability, and comparability of findings.

Although a three-level meta-analysis approach was used to account for variability and biases, the degree of heterogeneity across studies may limit the precision and generalisability of pooled estimates. Broad inclusion criteria, while beneficial for capturing a wide range of psychosocial interventions and outcomes, may have resulted in less precise or more variable estimates than if the studies were more homogenous. Additionally, collapsing the data into study-determined categories of psychosocial intervention and outcome may have oversimplified relationships, highlighting the need for more detailed subgroup analyses in future reviews.

This review focused on specific psychosocial outcomes following group intervention, underscoring the need for future reviews to explore additional aspects not covered in the current study, such as functional outcomes, neuropsychological impairments, language and communication, and social cognition. The heterogeneity in outcome measures identified in this review, including broadly defined and overlapping constructs, underscores the need for a more sophisticated and coordinated approach to measurement selection in psychosocial research. While most scales used in the included studies demonstrated reliability and validity, greater consistency in their application would allow for direct comparison of intervention efficacy. This inconsistency reflects the breadth in the conceptualisation of *psychosocial* functioning and its varied theoretical underpinnings. Future intervention studies could adopt established frameworks, such as those informed by the taxonomy of the International Classification of Functioning, Disability and Health (World Health Organization, 2013), which provide structured guidance for selecting outcome instruments in psychosocial research (e.g. Honan et al., 2019; Wilde et al., 2010). Additionally, group intervention studies should explicitly consider the theoretical mechanisms driving change, with social support emerging as a key candidate. Despite its well-documented role in promoting health and psychological adjustment (e.g. Uchino 2006; Wang et al., 2003), social support was assessed in only three of the 65 studies reviewed, each using a different measurement tool. Future research should further investigate social support as a mechanism of change and identify the most effective tool for its assessment, ensuring it can be harnessed more effectively in group interventions.

The focus of the current review on *group-based* interventions was deliberate and supported by the emerging evidence demonstrating the benefits that group-based belonging can provide. We found that 70% of studies included in this review referenced the benefits of the group modality and group process in intervention outcomes. However, none adequately measured or incorporated group processes into their designs. Social support, noted above, is only one potential mechanism. Other possible mechanisms of action include enhanced efficacy and perceived control, both identified as key bases for change in the social identity approach to health (Haslam et al., 2018). This underscores two important implications. First, future research that uses a group modality when delivering an intervention should include measures of group processes that are theorised mechanisms of change—such as treatment group identification which is what enables access to resources of support, self-esteem and control. Inclusion of these measures will help decipher the ‘active ingredients’ that underpin group intervention outcomes. Once we have a better understanding of these ingredients, we have the potential to use them in ways that amplify the efficacy of group interventions. Second, there is evidently a need for future group interventional studies in brain injury rehabilitation to understand the importance of group process and to integrate this into their design. This is especially the case in ABI populations, as participant engagement in group process may be undermined by their injury. This may even result in the extension of traditional rehabilitation beyond simply adapting to impairment, to promote post-traumatic growth (Grace et al., 2015; Ownsworth & Fleming, 2011).

## Conclusion

This is the first review to synthesise the literature on group psychosocial interventions in 3529 patients with ABI and demonstrate the potential for these programs to promote psychosocial outcomes in ABI survivors. The current state of evidence for integrating group psychosocial interventions into holistic rehabilitation is mixed, and the generalisability of results is limited by the quality of studies, inconsistent use of measurement tools, and heterogeneity in design and method. However, the focus of this review was on providing pragmatically useful findings for clinicians who, due to service realities, often treat patients with diverse injuries and backgrounds within the same group context. Insights into overall efficacy and differences between modalities therefore represent a valuable contribution to advancing clinical practice. The review also demonstrated that group processes as mechanisms of action are rarely considered and assessed in evaluations of intervention efficacy. Taking the next step to interrogate these processes more rigorously provides an opportunity to advance our understanding of the mechanisms of action in group-based interventions and ensure we optimise the delivery of psychosocial (and other) interventions for the benefit of the recipients.

## Data Availability

All the reviewed studies are publicly available.

## Code Availability

The data and analysis code for the meta-analysis are included as supplementary materials and are available via the Open Science Framework: https://osf.io/efbqk/?view_only=28e928d2a1e64e7f86dd03bfe423fc6d.
